# Molecular mechanism of the priming by jasmonic acid of specific dehydration stress response genes in Arabidopsis

**DOI:** 10.1186/s13072-016-0057-5

**Published:** 2016-02-24

**Authors:** Ning Liu, Zoya Avramova

**Affiliations:** School of Biological Sciences, University of Nebraska-Lincoln, Lincoln, NE 68588 USA

**Keywords:** Jasmonic acid priming, Dehydration stress memory, Superinduced memory transcription, MYC2, MEDIATOR, MED25, Stalled Pol II, Epigenetic marks, H3K4me3

## Abstract

**Background:**

Plant genes that provide a different response to a similar dehydration stress illustrate the concept of transcriptional ‘dehydration stress memory’. Pre-exposing a plant to a biotic stress or a stress-signaling hormone may increase transcription from response genes in a future stress, a phenomenon known as ‘gene priming’. Although known that primed transcription is preceded by accumulation of H3K4me3 marks at primed genes, what mechanism provides for their appearance before the transcription was unclear. How augmented transcription is achieved, whether/how the two memory phenomena are connected at the transcriptional level, and whether similar molecular and/or epigenetic mechanisms regulate them are fundamental questions about the molecular mechanisms regulating gene expression.

**Results:**

Although the stress hormone jasmonic acid (JA) was unable to induce transcription of tested dehydration stress response genes, it strongly potentiated transcription from specific ABA-dependent ‘memory’ genes. We elucidate the molecular mechanism causing their priming, demonstrate that stalled RNA polymerase II and H3K4me3 accumulate as epigenetic marks at the JA-primed ABA-dependent genes before actual transcription, and describe how these events occur mechanistically. The transcription factor MYC2 binds to the genes in response to both dehydration stress and to JA and determines the specificity of the priming. The MEDIATOR subunit MED25 links JA-priming with dehydration stress response pathways at the transcriptional level. Possible biological relevance of primed enhanced transcription from the specific memory genes is discussed.

**Conclusions:**

The biotic stress hormone JA potentiated transcription from a specific subset of ABA-response genes, revealing a novel aspect of the JA- and ABA-signaling pathways’ interactions. H3K4me3 functions as an epigenetic mark at JA-primed dehydration stress response genes before transcription. We emphasize that histone and epigenetic marks are not synonymous and argue that distinguishing between them is important for understanding the role of chromatin marks in genes’ transcriptional performance. JA-priming, specifically of dehydration stress memory genes encoding cell/membrane protective functions, suggests it is an adaptational response to two different environmental stresses.

**Electronic supplementary material:**

The online version of this article (doi:10.1186/s13072-016-0057-5) contains supplementary material, which is available to authorized users.

## Background

Different responses to an environmental stress by plants that have experienced an earlier encounter with the stress illustrate the concept of plant ‘stress memory’ [1–5]. Pre-exposing plants to a variety of pathogens, herbivory, or pre-treatment with biotic stress hormones, i.e., salicylic acid (SA) or jasmonic acid (JA), may result in higher resistance and stronger responses from defense-related genes in future attacks, a phenomenon known as ‘priming’ (called also ‘enhanced defense’) [6–8]. Likewise, plants that have experienced repeated cycles of dehydration/water recovery treatments maintain higher relative water content compared to plants encountering the stress for the first time, and a subset of dehydration stress responding genes displays a significantly altered transcriptional behavior [2, 9, 10]. Whole-genome transcriptome analyses of multiply stressed Arabidopsis plants revealed that 6579 genes responded to dehydration stress by either increasing or decreasing their transcription. Upon a repeated exposure, however, 1963 of the response genes produced significantly different amounts of transcripts compared to the amounts produced under the first stress. These genes defined the dehydration stress transcription ‘memory’ category in Arabidopsis; about 4500 genes provided similar transcriptional responses to each stress representing the ‘non-memory’ response category [9]. The operational criterion used for the term ‘transcriptional memory,’ therefore, is that transcriptional responses to similar stresses must be different.

Four distinct memory response types were recognized in multiply stressed *A. thaliana* and *Z. mays* plants, suggesting that transcriptional memory is an evolutionarily conserved mechanism that discriminates between a single and repeated stresses and modulates mRNA synthesis from the response genes accordingly [9, 10]. It is important to note also that GO analysis of encoded functions by the memory genes has indicated a biased functional distribution among the different memory response patterns suggesting biological relevance. By altering the transcript levels in a single and in multiple stresses, the memory genes alter the cellular responses and change the interactions between overlapping signaling pathways [9, 10].

A particular subset of about 320 response genes is upregulated by a first stress (S1) but produce significantly more transcripts during a second exposure (S2). These superinduced transcription memory genes encode proteins implicated in synthesis of protective, damage repairing, and detoxifying functions, like chaperones, dehydrins, osmolytes, and antitoxins. Presumably, their increased production helps to limit cellular and membrane damage under multiple stress exposures. For convenience, we have annotated the superinduced memory genes as [+/+] memory genes, where the first (+) sign indicates higher transcript levels produced in S1 relative to the initial prestressed watered (W) levels; the second (+) sign indicates that transcript levels in S2 are significantly higher than those in S1 [9].

JA-primed defense-related genes also increase their transcription upon a subsequent stress, a behavior that is consistent with transcriptional memory. However, the mechanism regulating priming may be different from the mechanism regulating the superinduced [+/+] transcriptional memory. For example, a pre-treatment with biotic stress-induced hormones (JA, SA/BTH) did not, or only slightly, induce transcription from defense genes but stimulate significantly their expression upon subsequent attacks [11–13]. In contrast, the superinduced transcription of the memory genes in S2 depends on active transcription that has occurred in S1 [2, 5].

At the chromatin level, however, the two events share a common feature illustrated by the presence of highly methylated (histone H3 tri-methylated Lys4, H3K4me3) nucleosomes uncoupled from active transcription. Altered chromatin structure and a variety of histone modifications have been observed in response to developmental, as well as to biotic and abiotic environmental cues, which have been extensively reviewed [14–18]. It is important to note that we make a distinction between a chromatin and an epigenetic mark to emphasize that a chromatin mark represents a modification that is dynamically associated (coupled) with the transcriptional process but is removed at the conclusion of that process. In contrast, a histone modification is considered an epigenetic (memory) mark if it persists longer than the stimulus establishing it; most importantly, it must affect the gene’s subsequent transcriptional behavior [2, 19–21]. According to this operational definition, H3K4me3 is an epigenetic mark for both the priming and the dehydration stress memory phenomena, as the H3K4me3 marks present during low-transcription phases are implicated in augmenting the subsequent transcription [2, 22]. A notable difference, however, is that elevated H3K4me3 at the dehydration stress response genes is retained as a ‘memory’ from the previous robust transcription [4], while at primed defense genes H3K4me3 accumulates before actual transcription [6, 22]. How H3K4me3 occurs in the absence of transcriptions is unclear. Furthermore, DNA-dependent RNA polymerase II phosphorylated at serine 5 of its C-terminal domain (Ser5P Pol II) is retained (stalled) at promoter-proximal regions of superinduced [+/+] memory genes during low-transcription recovery phase, contributing to their higher transcription in S2 [2, 23]. Whether Ser5P Pol II accumulates at JA-primed genes before dehydration stress-induced transcription has not been elucidated.

It is particularly important to note that physiological interactions between the JA- and ABA-mediated pathways, as well as co-regulated genes and shared components of the two signaling networks, have been well established [8, 24–26]. The basic helix–loop–helix (bHLH) transcription factor MYC2 is considered key participant in various signaling pathways including those regulated by JA and abscisic acid (ABA) [27, 28]. MYC2 is a major component of the core JA-signaling machinery. Its own activity is regulated by the presence/absence of bound repressor proteins (JAZs) and co-repressors (TPL or related TRPs) [29–31]. Removal of repressors by a JA-triggered proteolytic degradation of JAZs allows MYC2 to bind target promoters [30]. In order to initiate transcription from target genes, MYC2 recruits the MEDIATOR complex, which on its part recruits the components of the pre-initiation complex (PIC) to the promoters. Recruitment of MEDIATOR is achieved through physical interaction of MYC2 with the MED25 subunit of the plant complex [12, 32–34]. Whether a JA-MYC2-MED25-mediated mechanism functions also in gene priming events or whether a JA-signaled mechanism plays a role in the memory responses of dehydration stress/ABA-dependent genes has not been reported, to our knowledge. Here, we report that JA is able to prime transcriptional responses to dehydration stress in Arabidopsis and investigate possible molecular mechanisms for the JA-potentiated transcription of specific ABA-dependent genes.

## Results

### JA does not induce transcription from dehydration stress response genes but specifically primes a subset of memory genes

Based on the distinct transcriptional behavior displayed by the two related dehydration stress response genes *RD29B* and *RD29A* in response to a single and to a repeated dehydration stress, they were defined as a memory and non-memory response gene, respectively [2, 9]. Here, we use them as a prime model to study the effects of JA on their transcriptional responses to dehydration stress and to investigate the molecular mechanism of the JA-primed effects exerted specifically on *RD29B*.

**Fig. 1:**
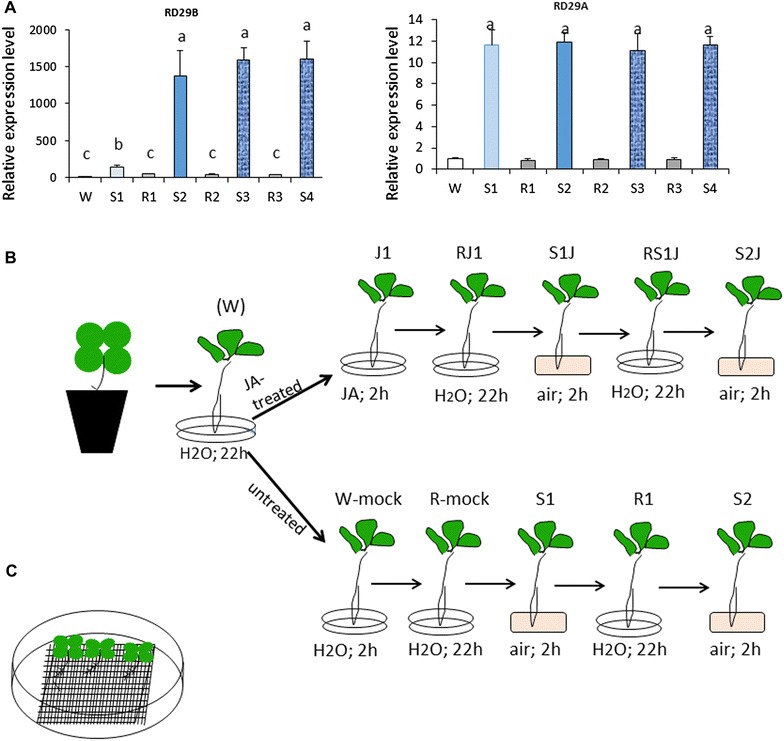
Transcription patterns of *RD29B* and *RD29A* under four rounds of dehydration stress and a diagram of the JA and dehydration stress treatments. **A** Transcript levels measured by real-time quantitative RT-PCR from *RD29B* and from *RD29A* genes after four cycles of exposure to dry air, annotated as dehydration stress treatments S1, S2, S3, and S4 separated by overnight periods of full recovery under water conditions annotated as R1, R2, and R3. Significantly elevated *RD29B* transcripts in S2, S3, and S4 compared to S1 illustrate the transcription memory response. Of note are the decreased transcript levels during water recovery (R1/R2/R3). All data represent results from three independent biological replicates; data shown indicate the mean ± SEM, *n* = 3. *Letters* above *error bars* indicate significant differences between stress responses (*p* < 0.05 according to Tukey’s multiple range test); *same letters*, or *no assigned letters*, indicate differences statistically not significant. *ACT8* was used as an internal control; **B** schematic illustration of employed treatments. *Letters* above *plantlets* annotate specific treatments; W-mock and R1-mock annotate treatments of control (JA-untreated) plants kept for 2 h and for 22 h in water, respectively, while experimental plants were undergoing JA/recovery treatment cycles; **C** illustration of plants grown and handled on meshes to avoid potential tissue damage

The transcriptional patterns displayed by *RD29B* and *RD29A* in response to a single (S1) and to multiple (S2, S3, S4) exposures to dehydration stress, separated by low-transcription recovery periods, are shown in Fig. 1A. Illustrating the transcriptional non-memory behavior, *RD29A* responded to each stress by increasing transcription to about the same degree, while returning to lower transcription during water recovery periods. In a stark contrast, *RD29B* produced significantly more transcripts in S1 than in W and significantly more in S2 than in S1, despite drastically lowering transcription between the stresses. These patterns illustrate the signature features of superinduced [+/+] transcriptional memory behavior. Specifically noted is that upon longer repeated exposures (S3 and S4) transcription from *RD29B* was still superinduced, at levels similar to those in S2. The ‘memory’ response, therefore, occurred after the first stress, was fully displayed in the second and was preserved during subsequent exposures to the stress (for length of the transcriptional dehydration stress memory, see [2]). Because memory is established during/after S1, we study the transcriptional responses displayed in S1 and in S2 to avoid potential caveats associated with developmental and/or senescence changes occurring during longer treatment periods.

Effects from JA upon the transcriptional responses to dehydrations stresses were investigated by the experimental design illustrated by the diagram in Fig. 1B. To limit effects from possible root or leaf damage during extraction from soil and/or during treatments, the plants were grown on meshes (Fig. 1C) and all manipulations were carried out by handling the meshes to avoid touching plant tissues. After removal from pots, the plants were left overnight under water conditions to recover. This treatment point is referred to as the initial prestressed watered state (W). JA-treated and JA-untreated samples were manipulated in parallel throughout the procedures, and their transcriptional activities were measured at the same time points during the day. During the 2-h exposure to JA (J1), the plants representing the untreated (control) sample were kept for 2 h in water (W-mock). Transcription from *RD29B* and *RD29A* in potted plants, after the initial overnight recovery from soil removal (W), during the 2-h (W-mock) treatment, or the following 22 h of water recovery (R-mock) was not affected (Additional file 1: Figure S1). From here on, (W) values represent transcript levels measured in the watered state after the initial overnight period following the removal from soil; all treatment points are annotated as shown in Fig. 1B.

**Fig. 2:**
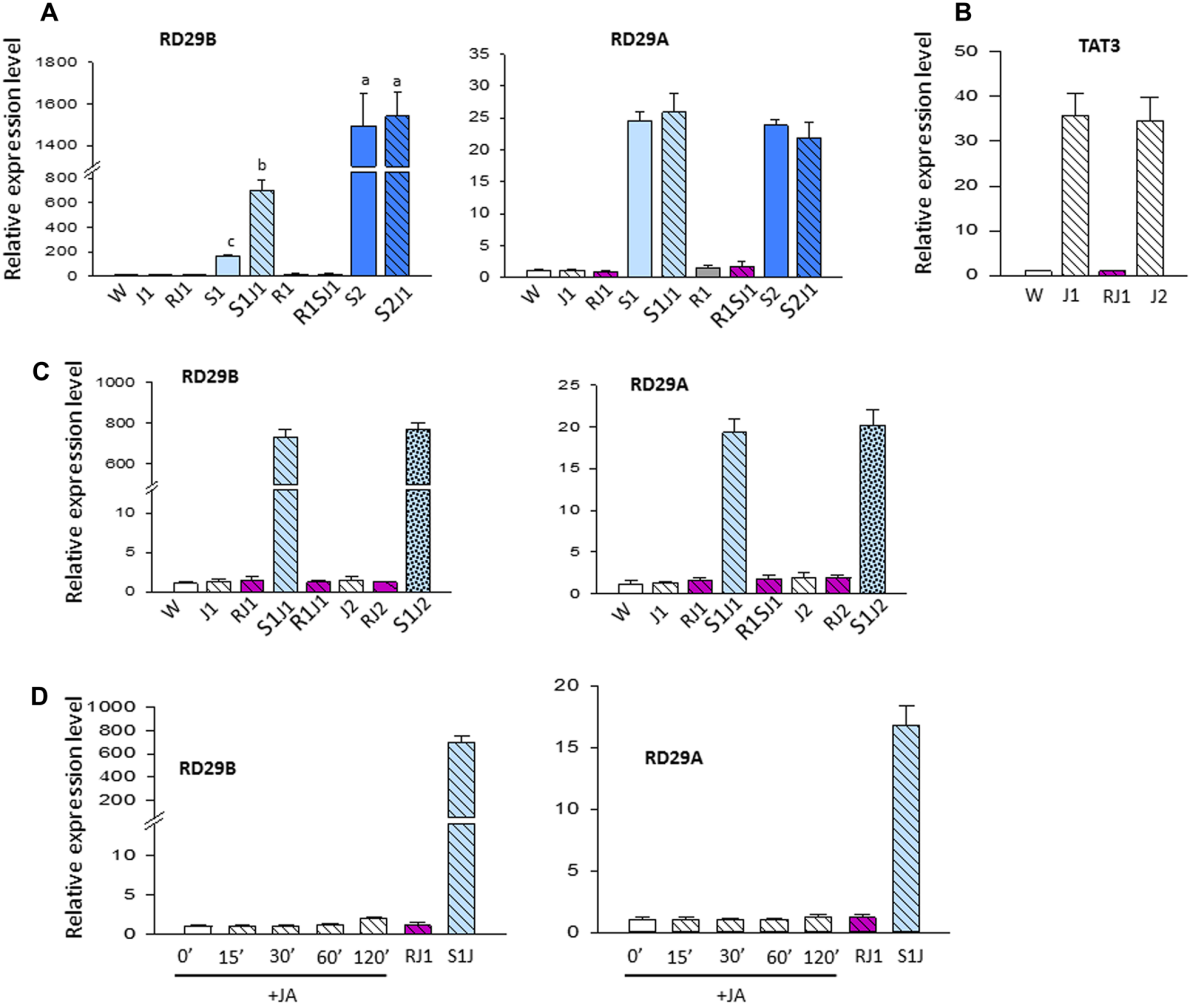
Transcription patterns of dehydration stress response genes *RD29B* and *RD29A* upon a repeated dehydration stress with or without JA-pre-treatment. **A** Transcript levels of *RD29B* and of *RD29A* measured by real-time quantitative RT-PCR in response to two consecutive exposures to dehydration stress separated by recovery R1 and the effects of the treatment with JA. From here on, annotations under columns indicate treatment points as specified in Fig. 1b. **B** Transcript levels from the marker JA-responsive gene, *TAT3,* in response to a single (J1) and repeated exposures to JA (J2). **C** Transcript levels from *RD29B* and of *RD29A* genes in response to dehydration stress after experiencing two exposures to JA (S1J1 and S1J2). **D** Transcript levels of *RD29B* and of *RD29A* genes during shorter periods of exposure to JA and after 22-h recovery in the absence of JA. From here on, data for JA-treated samples are represented by slanted columns. qPCR data were normalized against *ACT8* used as an internal control. Experiments were repeated three times, each with three qPCR measurements, and the results shown indicate the mean ± SEM, *n* = 3 replicates. *Letters* above the *error bars* indicate significant differences between stress responses (*p* < 0.05 according to Tukey’s multiple range test)

Treatment with JA under watered conditions did not induce transcription from either *RD29B* or *RD29A* (Fig. 2A). That JA was functionally active under these conditions was verified by the induced transcription from the marker JA-response gene *TAT3* used as a positive control (Fig. 2B).

After removal of JA and an overnight recovery under water (RJ1), the plants were subjected to a first dehydration stress (S1J1), followed by an overnight watered recovery (RS1J1), and a second dehydration stress (S2J1). Transcription from *RD29A* was not affected by the JA-treatment. However, there was a notable increase in *RD29B*’s transcript levels in response to the first dehydration stress in JA-treated, compared to untreated, plants (Fig. 2A) (significant difference, *p* < 0.05 according to Tukey’s multiple range test). Interestingly, the *RD29B* transcript amounts produced during the second stress of JA-treated plants (in S2J1) did not differ significantly from those of untreated plants (S2). The results suggested that JA specifically affected only the immediate subsequent transcription from *RD29B*. To determine whether JA had a similar potentiating effect on other dehydration stress response genes, we examined a few additional genes, namely *RAB18, LTP3,* and *LTP4* belonging to the same [+/+] memory category as *RD29B*, and *COR15* as a non-memory gene [2, 9]. None of these genes’ transcription was affected by JA under water conditions. In S1, however, the three memory genes produced significantly more mRNAs in JA-treated plants compared to the JA-untreated sample (Additional file 1: Figure S2A), while the transcript levels from *COR15* were similar in JA-treated and JA-untreated plants (Additional file 1: Figure S2B). Transcription in S2 was similar in JA-treated or JA-untreated plants.

The results suggested that JA affected (primed) transcription specifically from the memory genes in their response to the first dehydration stress. The superinduced responses in S2 were not affected.

### Increased transcription in S1 does not result from a delayed response to JA

To begin to understand how a JA-treatment affected transcription of ABA-dependent genes, we examined, first, whether elevated transcripts in S1J1 reflected, simply, a delayed transcriptional response to JA that was superimposed onto the subsequent dehydration stress response. The transcript levels measured after overnight recovery from the JA-treatment (RJ1), but before S1, were not increased providing no evidence for delayed transcriptional responses (Fig. 2A–C). A second round of JA-treatment (J2) did not induce transcription either, as the *RD29B* or *RD29A* transcripts in S1J2 did not increase in plants treated with JA twice (Fig. 2C). The transcriptional patterns from *RAB18, LTP3,LTP4*, and *COR15* confirmed those of *RD29B* and *RD29A*, respectively (Additional file 1: Figure S2A–D); the *TAT3* transcription was induced to a similar degree by a single (J1) or repeated (J2) treatments (Fig. 2B).

The possibility that the dehydration stress genes might have experienced an earlier induction of transcription that has been turned off by the 2 h of exposure to JA (the time at which J1 levels were routinely measured) was examined during shorter periods of JA exposures. Transcript levels measured from *RD29B* and *RD29A* after 15, 30, 60, 120 min, and the following 22-h recovery (RJ1) did not indicate significant changes (Fig. 2D), suggesting that JA did not induce significant transcriptional response until the implementation of the dehydration stress (S1J1). Thereby, neither repeated exposure nor delayed transcriptional responses to JA could account for the increased transcript levels from the tested memory genes in response to a subsequent dehydration stress. Possible molecular factors involved in this primed response were investigated next. We specifically note that as the priming is displayed in the response to the first dehydration stress, but not in the second, to understand how priming is achieved we focus on the events associated with the exposure to JA (J1), after recovery in the absence of JA (RJ1), and in the response to the first dehydration stress (S1J1). Furthermore, revealing transcriptional and chromatin factors involved in W-J1-RJ1 patterns of JA-treated plants and comparing them with those in W-S1-R1 of JA-untreated plants could provide insights into the common, as well as the distinct, factors involved in the subsequently enhanced transcriptions in S1J1 (for JA-treated) and in S2 (in JA-untreated plants), respectively.

### H3K4me3 at the dehydration stress response genes with and without exposure to JA

Increased transcription from primed defense genes has been linked to the occurrence of H3K4me3 established on their nucleosomes before the induction of transcription by a biotic stress [22]. It was important, then, to determine whether a JA-treatment would also cause accumulation of H3K4me3 at the dehydration stress response genes before S1. Chromatin immunoprecipitation (ChIP) assays with specific antibodies against H3K4me3 and three different gene regions of *RD29B* and *RD29A* were performed in parallel with chromatins isolated from JA-untreated and JA-treated plants.

**Fig. 3:**
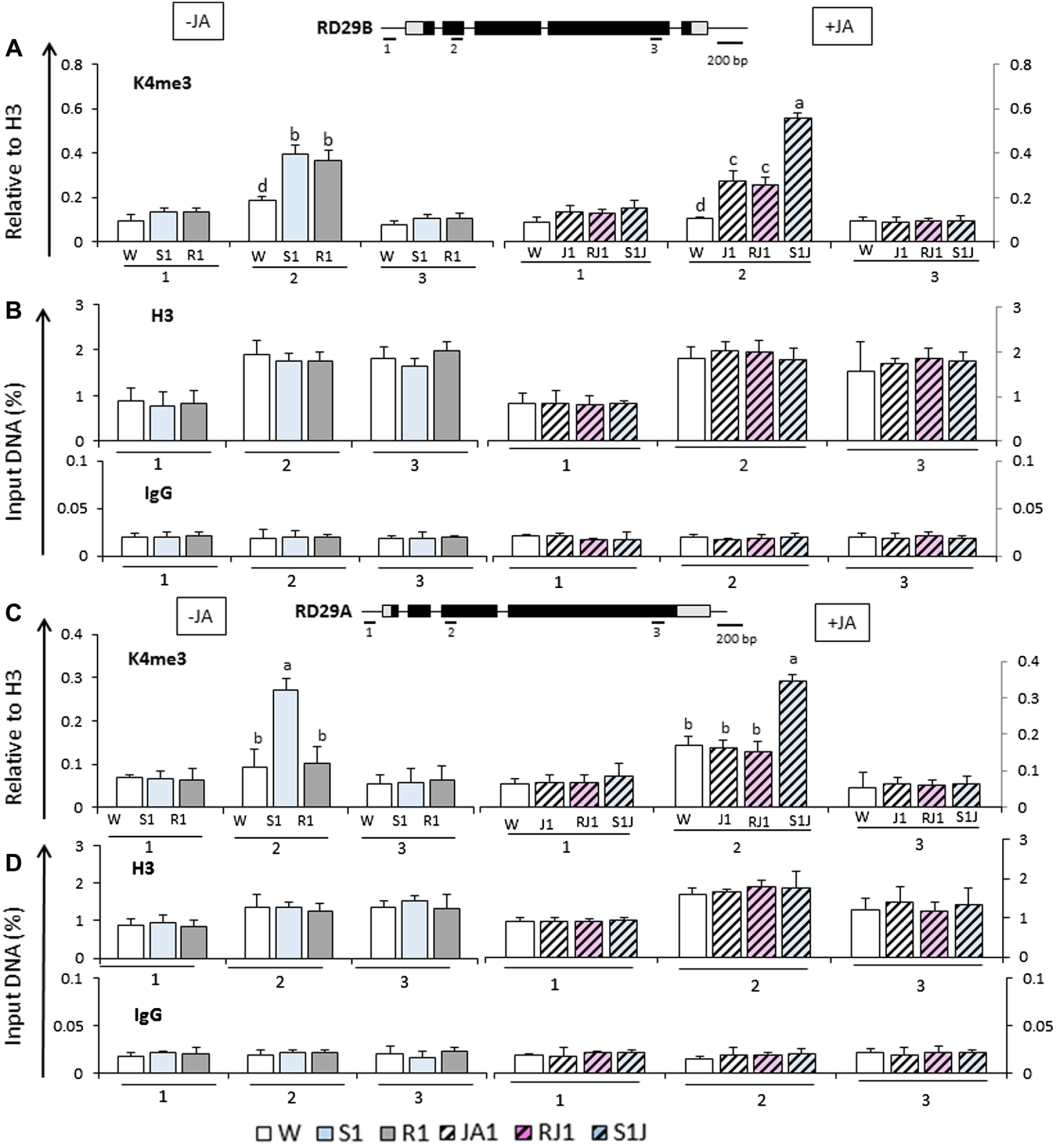
H3K4me3 and histone H3 distribution patterns at *RD29B* and *RD29A* genes in response to dehydration stress and to treatment with JA. The levels of histone H3K4me3 modifications and of histone H3 determined by ChIP–qPCR assays with specific antibodies under various treatments are annotated as indicated in Fig. 1B. **A** H3K4me3 levels at three different locations along the *RD29B* gene indicated by the gene diagram on *top*. H3K4me3 data were normalized versus values for histone H3 at the same locations. H3K4me3 levels before and after JA-treatments are shown in the same scale. **B** Histone H3 distribution determined by ChIP–qPCR with histone H3-specific antibodies and DNA recovered from the regions indicated in the gene diagram on *top*. ChIP–qPCR data from JA-treatments are shown in the same scale as those from untreated samples. ChIP–qPCR values obtained for IgGs at the specific amplicons are shown below; **C** H3K4me3 levels at *RD29A* at the regions indicated by the gene diagram on *top*. H3K4me3 data were normalized versus values for histone H3 at the same locations. Data from JA-treated and JA-untreated samples are shown in the same scale; **D** Histone H3 distribution determined by ChIP–qPCR with histone H3-specific antibodies and DNA recovered from the *RD29A* regions indicated in the gene diagram on *top*. ChIP–qPCR values obtained for IgGs at the specific amplicons are shown below. *Numbers* below *bars* indicate probed regions, as illustrated by the schematic diagram of the genes on *top*: promoter regions (1), untranslated regions (*gray box*), exons (*dark box*), and introns (*thin lines* between exons); region 2 is immediately downstream of TSS where the peak of K4me3 accumulates, and region 3 corresponds to downstream 3′-end sequences. Experiments were repeated three times, each with 3 RT-qPCR measurements, and the representative experiment shown indicates the mean ± SEM, *n* = 3 replicates. *Different letters* above *bars* indicate significant difference among the treatments in the region of interests (*p* < 0.05 according to Tukey’s multiple range test)

Without pre-treatment with JA, the H3K4me3 levels at both *RD29B* and *RD29A* significantly increased in S1 in response to the dehydration stress (Fig. 3A, C); together with the characteristic H3K4me3 peak displayed downstream of transcription start sites (TSS) (regions 2 in the gene models shown in Fig. 3 on top of panels A and C, respectively), these data were as expected and in full agreement with earlier results [35, 36]. The H3K4me3 levels were estimated relatively to the nucleosomal levels; histone H3 and IgGs distribution profiles at each amplicon are included in Fig. 3B, D. Of note is also the elevated presence of H3K4me3 remaining at *RD29B* (Fig. 3A) but not at *RD29A* (Fig. 3C) during the low-transcription period in R1, as it illustrates the specific retention of H3K4me3 at the memory genes functioning as an epigenetic mark to stimulate the subsequent transcription in S2 [2, 23].

Most important was the significant increase in H3K4me3 at *RD29B* in plants exposed to JA in water (in J1) before induction of transcription (region 2 in Fig. 3A). Moreover, elevated H3K4me3 persisted throughout the recovery period after the removal of JA (RJ1) and increased further after dehydration stress in S1J1. H3K4me3 was higher in S1J1 than in S1 (difference statistically significant, *p* < 0.05 according to Tukey’s multiple range test) consistent with higher transcription in S1J1. At *RD29A*, the H3K4me3 levels in J1 and RJ1 were similar to the levels in W (Fig. 3C) correlating with the lack of an effect from JA-treatment upon its transcription in S1J1.

An increase in H3K4me3 modified nucleosomes upon exposure to JA and retention of the H3K4me3 levels after the removal of JA was also found at the memory gene *RAB18* (shown in Additional file 1: Figure S3A), while no significant changes in H3K4me3 presence during these treatments were measured at *COR15* (Additional file 1: Figure S3C). H3K4me3 levels and distribution patterns during these treatments at the constitutively expressed *ACTIN7* genes are provided as an independent control (Additional file 1: Figure S4A).

Together, the results suggested that H3K4me3 accumulation at *RD29B* and *RAB18* after treatment with JA (in J1) and its retention during the following recovery (RJ1) contributed to their subsequently increased transcription in S1J1; unchanged H3K4me3 levels at *RD29A* and *COR15* after J1 and RJ1 reflected the similar-level transcription displayed in S1 and in S1J1. It is relevant here to emphasize the parallel between the elevated presence of H3K4me3 at the memory genes in R1 and its occurrence in J1-RJ1 after exposure to JA as H3K4me3 emerges as the common chromatin feature underlying the increased subsequent transcription in S2 and in S1J1, respectively. However, in JA-treated plants H3K4me3 appears before S1, while in R1 of JA-untreated plants H3K4me3 is retained after S1, suggesting that the mechanisms responsible for higher H3K4me3 levels in R1 and in J1 are, most likely, different. The immediate question, then, was how H3K4me3 accumulated at primed genes in J1 before the induction of transcription.

### Ser5 Pol II at JA-primed dehydration stress memory response genes

**Fig. 4:**
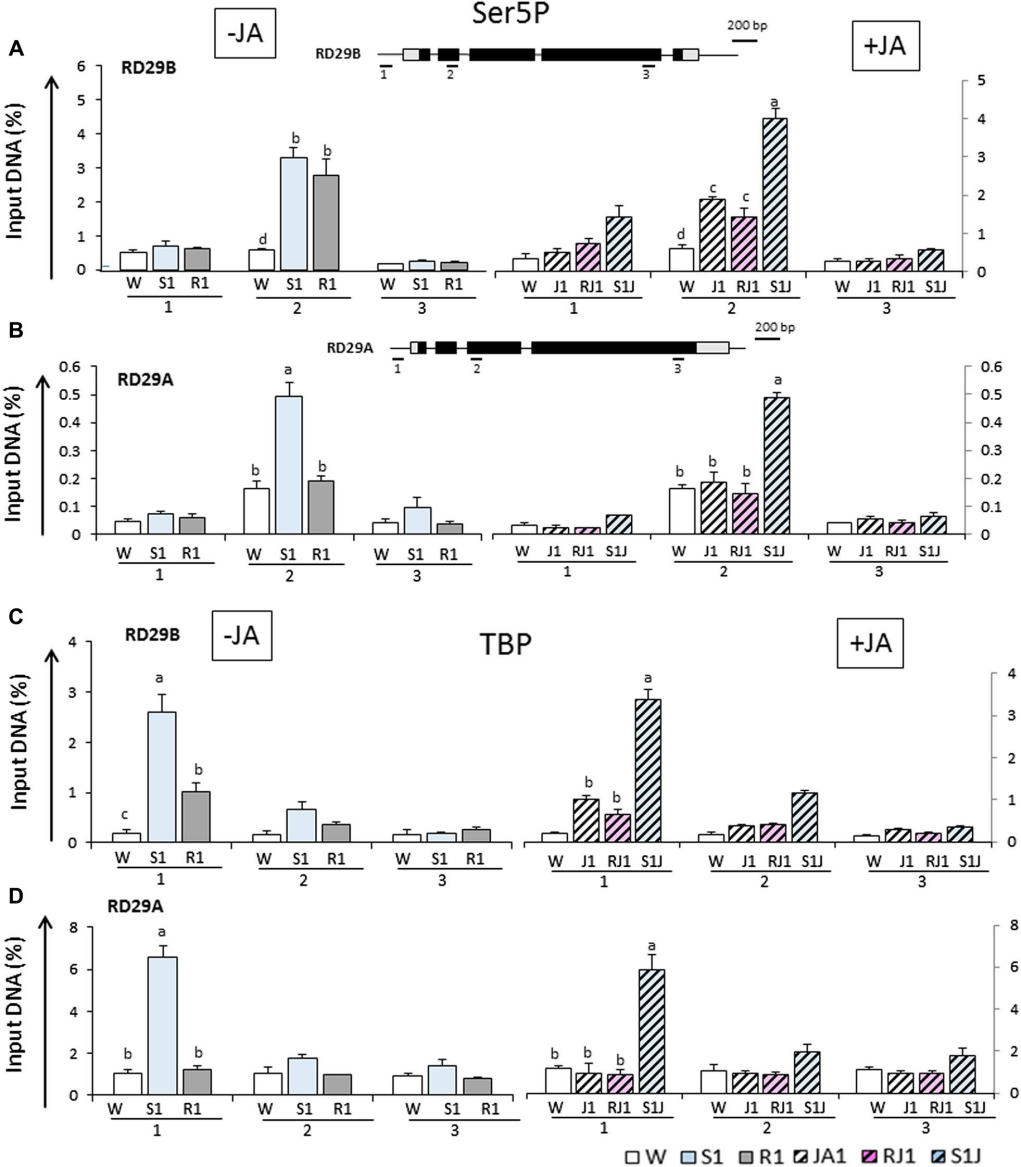
Ser5P PolII and TATA binding protein (TBP) distribution profiles at *RD29B* and *RD29A* genes in response to dehydration stress and to treatment with JA. Ser5PPol II and TBP levels measured by ChIP–qPCR assays with specific antibodies under the various treatments, as annotated in Fig. 1B. Schematic diagrams of the genes are shown on *top* and annotated as in Fig. 3 above. *Numbers* below *bars* indicate regions assayed by ChIP. **A** Ser5 Poll II levels measured by ChIP–PCR at the indicated regions of *RD29B*; **B** Ser5 Poll II levels measured by ChIP-PCR at the indicated regions of *RD29A*; **C** distribution and TBP levels determined by ChIP-PCR at *RD29B*; **D** distribution and TBP levels determined by ChIP-PCR at *RD29B. Numbers* below show the locations of the regions analyzed by the ChIP assays. Experiments were repeated three times, each with three RT-qPCR measurements, and the representative experiment shown indicates the mean ± SEM, *n* = 3 replicates. For each point, *letters* above *bars* indicate significant difference among the treatments in the regions of interest (*p* < 0.05 according to Tukey’s multiple range test)

Presence of promoter-proximal Ser5 Pol II is considered responsible for histone modifications at the 5′-ends of yeast, animal, and Arabidopsis genes [35, 37, 38]. It was critical, then, to determine whether Ser5 Pol II accumulated at primed genes in a JA-dependent manner as a potential mechanism for the occurrence of H3K4me3. ChIP assays were performed with specific antiSer5 Pol II antibodies in JA-treated or JA-untreated plants, before and after dehydration stress-induced transcription. In JA-untreated plants, the dehydration stress-increased transcription (in S1) correlated with increased Ser5-modified Pol II at the 5′-ends of both *RD29B* and *RD29A*, with a peak downstream of the TSS (region 2 in the gene models in Fig. 4A, B). In full agreement with earlier results [2], Ser5 Pol II remained elevated at *RD29B* during the gene’s low-transcription recovery period (R1) (Fig. 4A), while Ser5 Pol II levels at *RD29A* decreased in R1 correlating with the decreased transcription (Fig. 4B). The important new observation here was that after exposure to JA, the Ser5Pol II levels at *RD29B* significantly increased in J1 before induction of transcription (Fig. 4A). Moreover, the Ser5Pol II levels remained elevated during the 22-h recovery after removal of JA (R1J1) and increased further in S1J1. Ser5Pol II signal in S1J1 was slightly higher than in S1 but Tukey’s multiple range test defined it as statistically significant, *p* < 0.05. ChIP signals measured from downstream region 3 provided background signal levels. JA-treatment did not affect Ser5Pol II levels at *RD29A* consistent with its unchanged transcription by the exposure to JA (Fig. 4B). Higher presence of Ser5Pol II in R1 (after S1) in JA-untreated plants, as well as in J1 and RJ1 (in JA-treated plants), were found also at *RAB18* (Additional file 1: Figure S5A) but not at *COR15* (Additional file 1: Figure S5B), confirming the distinct Ser5Pol II accumulation patterns at JA-primed versus not-primed dehydration stress response genes. The Ser5Pol II patterns at the constitutively transcribed *ACTIN7* gene are shown as an independent control in Additional file 1: Figure S4B.

It is plausible, then, that the JA-caused accumulation of Ser5Pol II at the primed genes is responsible for the H3K4me3 occurrence before the induced transcription. The overlapping profiles of Ser5Pol II and H3K4me3 levels throughout the treatments support with such a conclusion.

### JA stimulates recruitment of TBP to the promoters of the memory genes

An immediate question was what mechanism(s) caused the JA-triggered accumulation of Ser5Pol II at the primed genes before the activation of their transcription. The critical step in transcription initiation is the formation of the pre-initiation complex (PIC), which involves recruitment of the TATA binding protein (TBP) to target promoters [39, 40]. To establish whether JA-treatment caused PIC assembly at the dehydration stress response genes’ promoters, we performed ChIP assays with specific antibodies against TBP under conditions, when priming was established.

Low TBP at the *RD29B* promoter in the initial prestressed state (W) correlating with low transcription, and elevated presence of TBP in S1 reflecting the increased transcription during dehydration stress (Fig. 4C) were measured in region 1 corresponding to the promoter regions (see gene model in Fig. 4A). Interestingly, the TBP levels remained elevated during the water recovery (R1), suggesting that the basal transcriptional machinery did not fully disassemble during R1. Furthermore, exposure to JA also resulted in increased TBP at the *RD29B* promoter in J1 (differences with levels in W statistically significant, *p* < 0.05 according to Tukey’s multiple range test) and remained elevated during the 22-h recovery after the removal of JA (RJ1). Increased transcription in response to the dehydration stress (in S1J1) correlated with an increase in TBP levels (Fig. 4C). The TBP signal from downstream gene regions provides an internal control for background levels. TBP levels increased also at the *RD29A* promoter when its transcription was induced by the dehydration stress but did not change in J1 (Fig. 4D).

To understand how TBP appeared at *RD29B* after the exposure to JA in the absence of transcription, we examined a possible role for the MEDIATOR complex.

### MED25 facilitates the transcription of both memory and non-memory dehydration stress/ABA-response genes

The MEDIATOR complex is the bridge between transcription factors, PIC, and Pol II [39, 41, 42]. In plants, the MEDIATOR complex, via its subunit MED25, has been implicated in JA-mediated stress responses [12, 32–34, 43]. Here, we investigated whether MEDIATOR, via MED25, was involved in depositing the transcriptional machinery to the promoters of JA-primed dehydration stress/ABA-dependent genes. First, we examined whether MED25 was involved in the transcription and/or the priming of dehydration stress response genes.

**Fig. 5:**
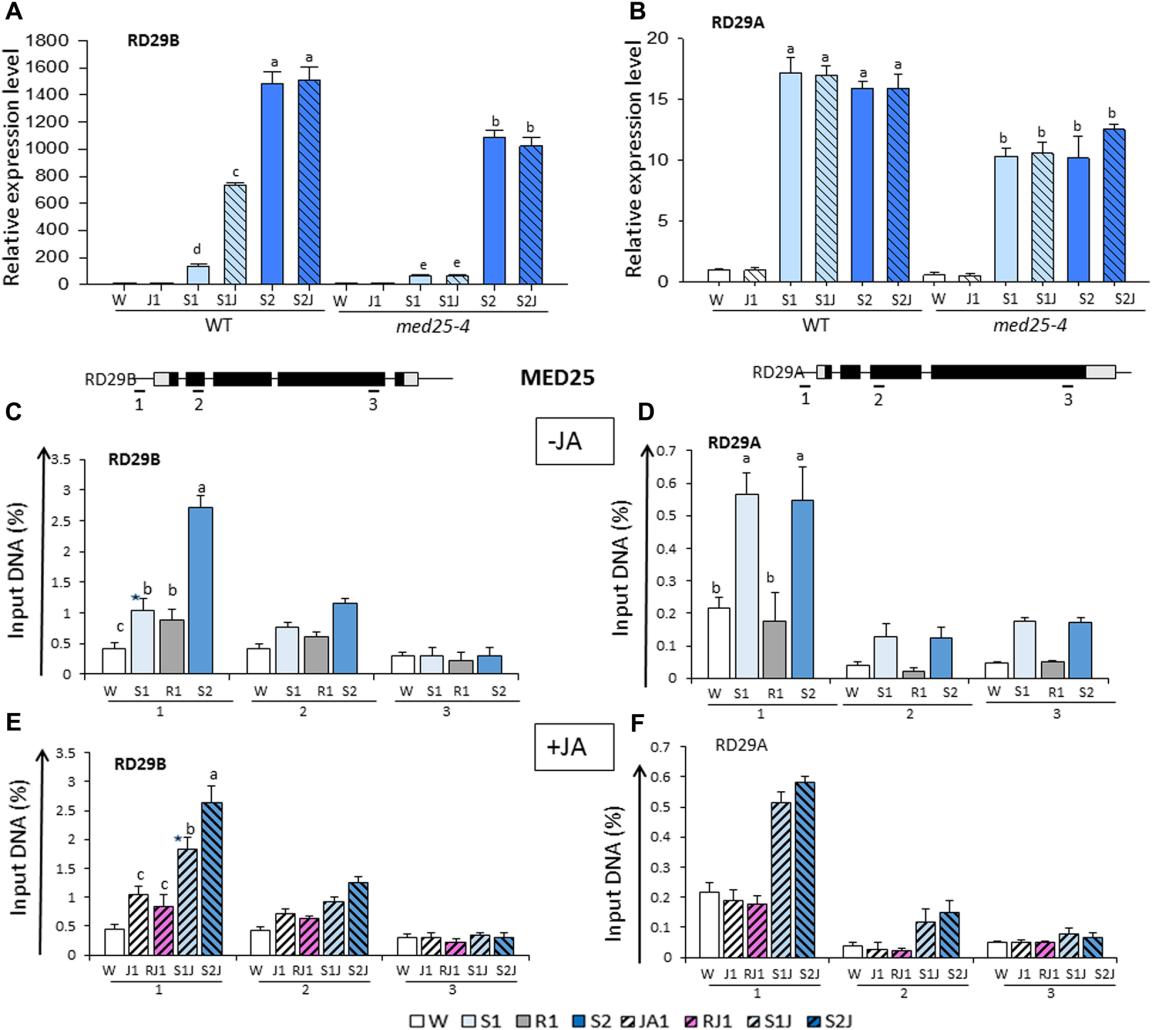
Transcriptional patterns and MED25 distribution profiles at *RD29B* and *RD29A* genes under dehydration stress with and without JA-treatment. **A**, **B** Transcript levels of *RD29B* and of *RD29A* genes measured by real-time quantitative RT-PCR in wild type and *med25* mutant backgrounds under the various treatment conditions, as indicated. *ACT8* was used as an internal control for normalization; **C**, **D** distribution profiles of HA-tagged MED25 in plants without exposure to JA determined by ChIP–qPCR assays with anti-HA antibodies and gene sequences from probed regions indicated by the *numbers* below on the gene structure diagrams shown on *top*; **E**, **F** distribution profiles of MED25 measured by ChIP–qPCR assays with anti-HA antibodies and gene sequences from *RD29B* and at *RD29A*, respectively, in JA-treated plants. Data shown are the average of three independent biological replicates; *error bars* represent ± SEM, *n* = 3. For each point, *letters* above *bars* indicate significant difference among the treatments in the regions of interest (*p* < 0.05 according to Tukey’s multiple range test). *Asterisks* above the *bars* in **C**, **E** indicate significant differences in MED25 levels between S1 and S1J, respectively, according to Student’s *t* test (*p* < 0.01)

**Fig. 6:**
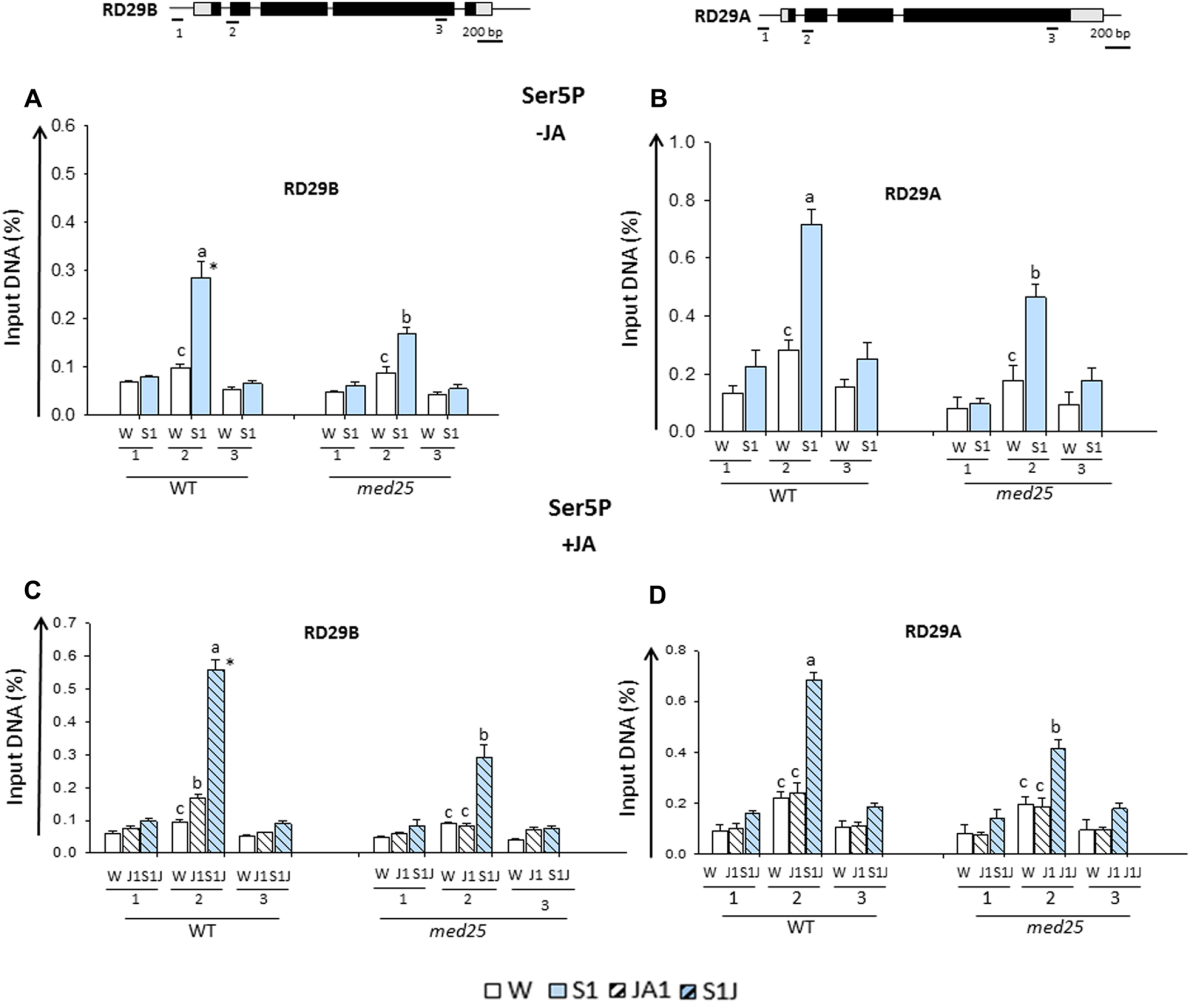
Ser5Pol II levels at *RD29B* and *RD29A* in response to dehydration stress S1 in the absence of a functional MED25. Distribution and levels of Ser5Pol II at *RD29A* and *RD29B* determined by ChIP–qPCR assays with specific antibodies and amplified regions numbered as indicated on *top*. **A**, **B** Ser5Pol II levels at *RD29B* and *RD29A* measured in W and in S1 from wild type and *med25* mutant plants without exposure to JA, respectively; **C**, **d** Ser5Pol II levels at *RD29B* and *RD29A* in W and in S1 from wild type and *med25* mutant plants, respectively, in response to the treatment with JA. Experiments were repeated at least three times, each with three replicates, and the representative experiments shown indicate the mean ± SEM, *n* = 3 replicates. *Letters* above *bars* indicate significant difference among the treatments in the regions of interest (*p* < 0.05 according to Tukey’s multiple range test). Significant differences in Ser5Pol II levels in JA-untreated (S1) and JA-treated (S1J) samples are indicated by *asterisks* in **A**, **C**, according to Student’s *t* test

In *med25* mutant background, the *RD29B* transcript levels were significantly downregulated in both S1 and S2 compared to wild type (Fig. 5A) implicating MED25 in the responses to the first and the second stress. However, transcription in S2 was still significantly higher than in S1, indicating that the memory response was not eliminated in *MED25*-deficient plants. Specifically noted is that transcription from *RD29A* was also downregulated in *med25* mutants (Fig. 5B) implicating MEDIATOR in its transcription as well. Therefore, the MEDIATOR complex contributed to the transcriptional responses in the single and in the repeated dehydration stresses from both *RD29B* and *RD29A* genes. A requirement for MED25 in the transcription of dehydration stress-related genes was confirmed also by the decreased transcription from *RAB18, LTP3*, *LTP4*, and *COR15* genes in the *med25* background (Additional file 1: Figure S6). In addition, the transcription from *RD29B* and the other memory genes (*RAB18, LTP3*, and *LTP4*) in *med25* mutants was not enhanced after the treatment with JA (in S1J1) (Fig. 5A, Additional file 1: Figure S6A), indicating that a functional MED25 was required for their JA-primed transcription in response to dehydration stress. Transcription from *RD29A* and *COR15* remained lower but unaffected by the treatment with JA in *med25* mutant plants (Figs. 5B, 6B).

The next important question was whether MED25 functioned directly at the promoters of the genes when responding to a dehydration stress, without pre-treatment with JA. Transgenic plants expressing the HA-tagged MED25 fusion protein were analyzed by ChIP assays with antibodies against HA-tagged MED25. Higher presence of MED25 was found at the *RD29B* promoter (region 1) in S1 compared to the levels before the stress (W) (Fig. 5C). Interestingly, MED25 levels remained elevated during the recovery phase (R1), suggesting that it did not dissociate from the *RD29B* promoter after S1. MED25 increased also at the *RD29A* promoter during dehydration stress-induced transcription but its presence decreased when *RD29A* transcription decreased in R1 (Fig. 5D). Signals from regions at the 3′-ends of the genes provide background HA-MED25 signal levels. Together, the results indicated that MED25 was recruited to *RD29B* and *RD29A* promoters by a dehydration stress/ABA-activated mechanism. As it was unknown whether MED25 was involved also in the transcriptional response to the second stress, we determined MED25 levels in S2. In JA-untreated plants, significantly higher MED25 was found in S2 compared to S1 at *RD29B* (Fig. 5C) but not at *RD29A* (Fig. 5D) suggesting an involvement of MED25 in the superinduced transcriptional response of the memory gene.

Next, we asked whether the JA-mediated priming pathway was also involved in recruiting MED25 to the promoters of the dehydration stress/ABA-dependent genes. To answer this question, ChIP assays in transgenic plants stably expressing the HA-MED25 fusion protein were performed with specific anti-HA antibodies under the different treatment conditions.

Higher MED25 levels at the *RD29B* promoter were displayed after the treatment with JA (in J1) (Fig. 5E). MED25 remained elevated also during the 22-h recovery (RJ1) in the absence of JA and increased further upon activation of the ABA-pathway in S1J1 (Fig. 5E). MED25 levels in S1J1 were higher than in S1 (differences statistically significant, *p* < 0.05 according to Tukey’s multiple range test). Upon a second stress, however, the MED25 levels were similar in S2J1 and in S2, consistent with the similar-level transcription of JA-treated and JA-untreated plants in S2. No significant changes in MED25 levels occurred at the *RD29A* promoter after exposure to JA under water but the levels significantly increased upon the dehydration stress, supporting an activating function for the MEDIATOR complex in *RD29A*’s transcription. Higher initial levels of MED25 at *RD29A* (in W) reflect, most likely, the fact that a basal level transcription of *RD29A* is taking place under non-stressed watered conditions [44].

MED25 appeared also at the promoters of *RAB18, LTP3, LTP4*, and *COR15* in response to S1 but was found only at *RAB18, LTP3,* and *LTP4* in response to JA (Additional file 1: Figure S7A-B).

Collectively, the results indicated that a dehydration stress/ABA-dependent mechanism recruited MED25 to the promoters of the tested genes in response to dehydration stress. Although less efficiently, transcription still occurred in the *med25* background, suggesting that the role of MED25 is mainly to facilitate their transcription. MED25 is recruited to dehydration stress responding genes by two separate mechanisms: one initiated by a dehydration stress/ABA-mediated pathway, while the other is JA-regulated recruiting MED25 only to specific dehydration stress genes’ promoters.

### MEDIATOR and Ser5P Pol II at the dehydration stress/ABA-response genes

The MEDIATOR complex and RNA Pol II interact both functionally and physically in PIC and MEDIATOR enhances the TFIIH-dependent phosphorylation of the Pol II CTD in yeast and animal transcription systems [41, 42, 45, 46]. Whether MEDIATOR plays a role in establishing Ser5P Pol II at Arabidopsis genes has not been clear.

To determine whether accumulation of Ser5PPol II depended on MED25, we analyzed the effects from a MED25′s depletion on Ser5PPol II at its peak position in response to S1. ChIP assays with antiSer5Pol II antibodies and sequences from the respective regions (indicated as regions 2 on the gene models in Fig. 6) of *RD29B* and *RD29A* in wild type and in *med25* mutant backgrounds were conducted. Significantly lower Ser5P Pol II was measured in S1 at both *RD29B* and *RD29A* genes in the *med25* background compared to wild type (Fig. 6A, B). The results suggested MED25 was required for establishing Ser5P Pol II at the genes during their dehydration stress-induced transcription, in full agreement with the above conclusion that a dehydration stress/ABA-dependent mechanism recruits MED25 to the response genes.

The question of whether a MED25 deficiency would affect the JA-induced Ser5P Pol II accumulation at the genes before the stress and in response to S1 was examined in in *med25* mutants in parallel with wild-type plants. Consistent with the results above, treatment of wild-type plants with JA increased Ser5P PolII presence at *RD29B* (region 2) before dehydration stress (in J1) and the Ser5P Pol II levels further increased with the induction of transcription in S1J1 (Fig. 6C). Exposure of *med25* mutants to JA, however, did not result in a Ser5PPol II increase before the dehydration stress (levels in J1 similar to prestressed levels in W). In *med25* plants, the Ser5P Pol II levels in S1J1 were not significantly different from the levels in S1 of the wild type (Fig. 6A, C) consistent with a conclusion that primed transcription was eliminated in the *med25* background. The Ser5PPol II levels and distribution patterns at *RD29A* in *med25* mutants also agreed with the lower transcription in S1 and with the lack of JA-dependent priming effects (Fig. 6B, D).

Therefore, MEDIATOR (MED25) is required for the Ser5P Pol II accumulation at *RD29B* and *RD29A* genes and for their dehydration stress-activated transcription in S1. However, elevated presence of Ser5P Pol II before the dehydration stress depended on the JA-mediated recruitment of MEDIATOR specifically to the promoter of *RD29B*. How specificity was achieved was studied next.

### The transcription factor MYC2 and the priming of dehydration stress response genes

The mechanism determining the promoter specificity of the MEDIATOR complex is based on specific interactions of its subunits with DNA-binding transcription factors [45, 47, 48]. In particular, the JA-regulated TF MYC2 binds specifically the MED25 subunit and recruits the MEDIATOR complex to JA-dependent defense-related genes [12, 32, 33]. To find out whether MYC2 was the factor linking the JA-initiated-specific priming of the memory dehydration stress genes, we examined, first, whether MYC2 had any effect on the transcriptional responses of dehydration stress associated genes.

**Fig. 7:**
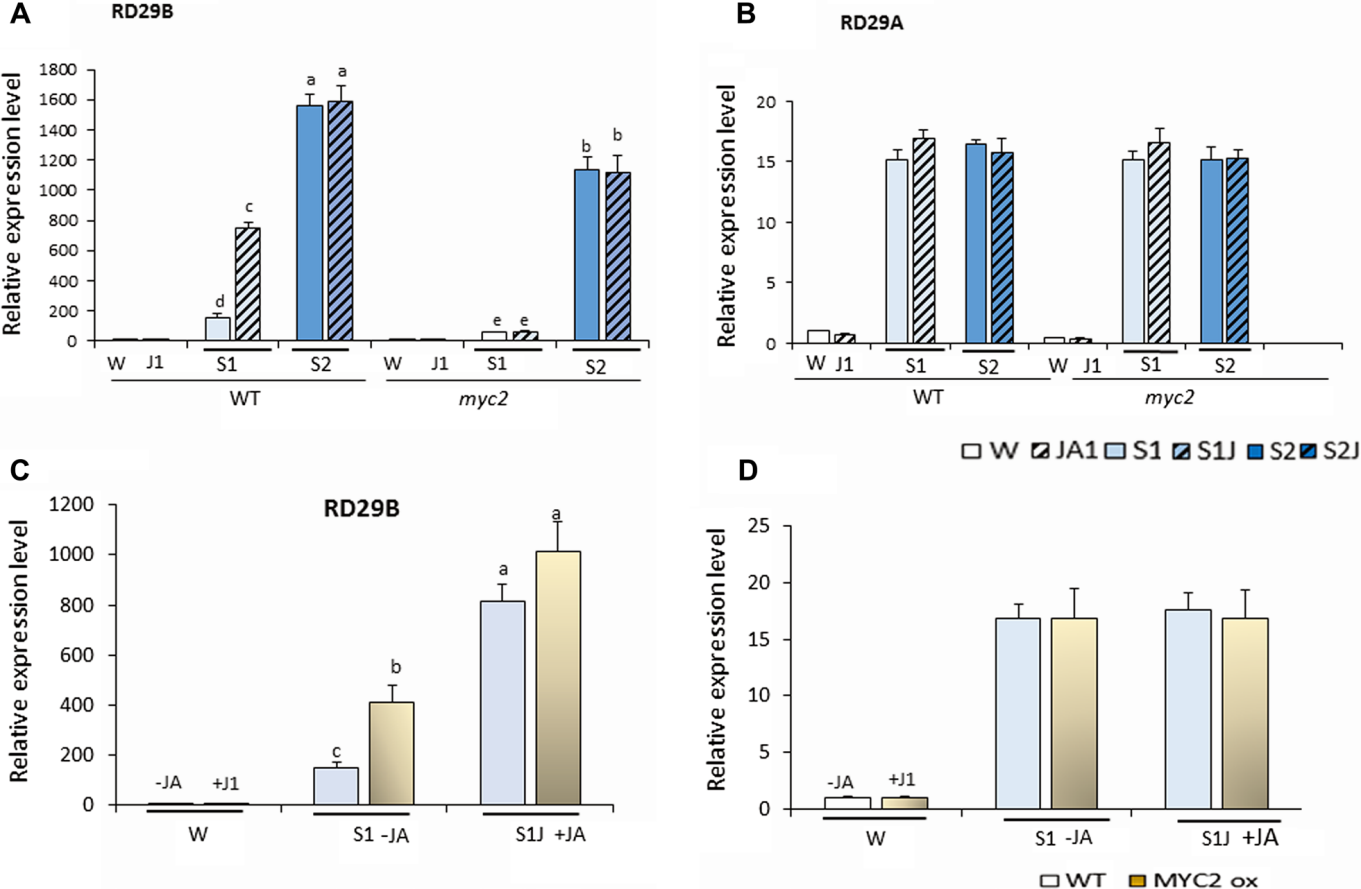
Transcriptional patterns of *RD29B* and *RD29A* in *MYC2*-deficient and *MYC2*-overexpressing plants in response to dehydration stress and exposure to JA. **A**, **B** Transcript levels of *RD29B* and of *RD29A* in S1 and in S2 measured by real-time quantitative RT-PCR in wild type and in *myc2* mutant plants without treatment with JA and after pre-exposure to JA; **C**, **D** transcript levels of *RD29B* and of *RD29A* in W and in S1 of wild type and of *MYC2*-overexpressing plants without treatment with JA and after pre-exposure to JA. *ACT8* was used as an internal control for normalization. Results were mean from three independent biological replicates; *error bars* represents the ± SEM, *n* = 3. *Letters* above *bars* indicate significant difference in transcript levels among the respective drought treatments (*p* < 0.05 according to Tukey’s multiple range test)

In *MYC2*-deficient plants, the transcript levels from *RD29B*, but not from *RD29A*, were significantly lower in both S1 and in S2 compared to wild type (Fig. 7A, B). MYC2 was also involved in the S1 and S2 transcription from *RAB18, LTP3*, and *LTP4*, but not from *COR15* (Additional file 1: Figure S8), suggesting that MYC2 contributed to the transcriptional responses specifically of the memory genes. As transcription from *NCED3*, critical for ABA synthesis [49] and of the ABF genes (*ABF2*, *ABF3*, and *ARF4*) encoding the ABRE-binding factors essential for dehydration stress response genes’ expression [50] was not affected by the lack of MYC2 [21] (Additional file 1: Figure S9), the lower transcription in the *myc2* background, most likely, did not result from downregulated synthesis of ABA or of the ABFs.

Whether MYC2 affected the JA-induced priming of the memory genes was also examined. In a stark contrast to wild type, treatment of *myc2* mutants by JA did not enhance the *RD29B* transcription in S1J1 (Fig. 7A); the primed responses from the other memory genes were also lost in the *myc2* background (Additional file 1: Figure S8). Therefore, a functional MYC2 is required for the JA-dependent enhanced transcription of primed genes in S1J1. The transcription from *RD29A* and *COR15* was not affected in *myc2* mutants after JA-treatment (Fig. 7B, Additional file 1: Figure S8) consistent with the lack of JA-mediated effects on their transcription.

### Overexpressed MYC2 primes dehydration stress memory genes without stimulation by JA

The requirement for a functional MYC2 to achieve the JA-dependent priming of the memory genes is consistent with current understanding that a JA-initiated mechanism activates MYC2, allowing it to bind target promoters [30]. It was important to establish, then, whether a constitutively present MYC2 would enhance the transcription of the genes in S1 without pre-treatment with JA. To answer this question, we analyzed the *RD29B* and *RD29A* transcript levels in transgenic plants overexpressing MYC2 under the constitutive 35S promoter. Overexpressed MYC2 (OX-MYC2) had no impact on *RD29B* transcription under water conditions (Fig. 7C). Upon dehydration stress, however, transcription from *RD29B* was higher in the OX-MYC2 genotype compared to the wild type (difference statistically significant, *p* < 0.05 according to Tukey’s multiple range test). Treatment with JA resulted in a stronger increase in transcription in S1J1. The increase in transcript levels in S1J1 was similar in wild type and in OX-MYC2 plants (Fig. 7C). No significant differences were measured for *RD29A* (Fig. 7B, D). Thereby, constitutively expressed MYC2 is capable of increasing transcription in response to a subsequent dehydration stress, mimicking to some degree the JA-induced priming. However, the priming effects were stronger when the JA-signaling pathway was activated by the exposure to JA.

### MYC2 and Ser5P Pol II accumulation at dehydration stress response genes

**Fig. 8:**
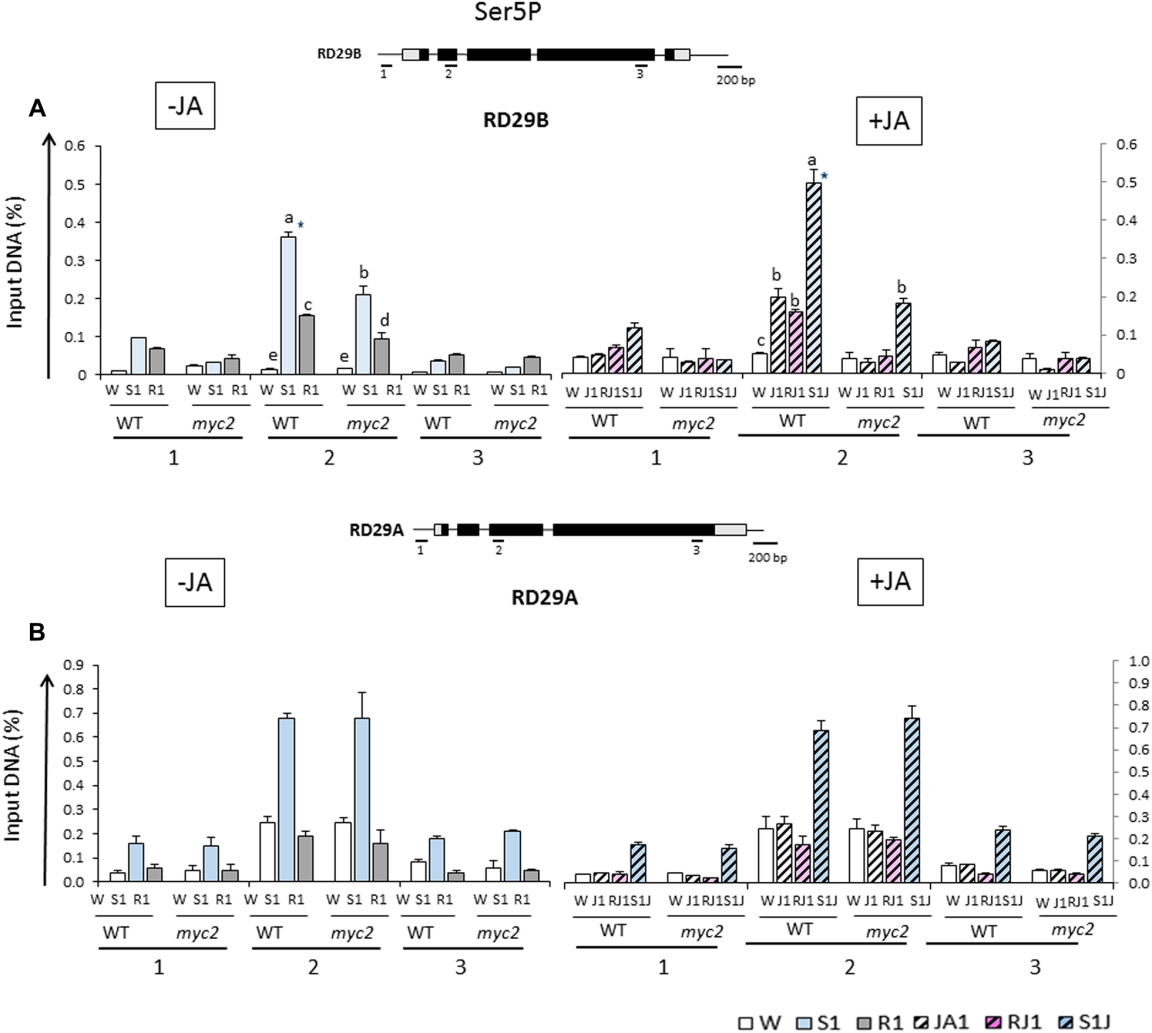
Ser5Pol II distribution profiles at *RD29B* and *RD29A* in *myc2* mutants under stress treatments. **A** Ser5Pol II levels at *RD29B* measured by ChIP–qPCR in wild type and *myc2* backgrounds with specific antibodies and probed regions indicated by the *numbers* below *bars* as indicated on the gene model on *top*; **B** Ser5Pol II levels at *RD29A* determined by ChIP–qPCR in wild type and *myc2* backgrounds with specific antibodies and probed regions indicated by the *numbers* below *bars* as indicated on the gene model on *top*. Experiments were repeated three times, each with three replicates, and the representative experiments shown indicate the mean ± SEM, *n* = 3 replicates. *Letters* above *bars* indicate significant difference among the treatments in the regions of interest (*p* < 0.05 according to Tukey’s multiple range test). Significant differences in Ser5 Pol II levels at *RD29B* in JA-untreated (S1) and JA-treated (S1J1) samples are indicated by *asterisks* in **a** according to Student’s *t* test

That MYC2 is the factor linking the JA-potentiated priming of *RD29B* with the accumulation of the activated Ser5P-modified Pol II at the 5-ends of the dehydration stress response genes was confirmed by ChIP assays with antiSer5P Pol II antibodies in *myc2* mutants before, and after, treatments with JA. Compared to the levels in wild type, the presence Ser5P Pol II in S1, at the peak of its accumulation (region 2) of *RD29B*, was significantly lower in *myc2* mutants (Fig. 8A). Furthermore, Ser5P Pol II in *myc2* mutants did not increase under JA before the dehydration stress (Fig. 8A). We conclude that MYC2 mediates the JA-priming effects by contributing to the Ser5P Pol II accumulation before dehydration stress. The Ser5P Pol II levels and distribution at *RD29A* were not significantly affected by MYC2 deficiency or by the treatment with JA (Fig. 8B).

### MYC2 binds to the promoter of *RD29B* in response to JA and to dehydration stress in S1 but not in S2

Lastly, we investigated whether MYC2 affected directly the JA-mediated transcriptional responses by examining specifically the promoter regions (regions 1) of *RD29B* and *RD29A* for possible association with MYC2 in response to dehydration stress and/or JA. Transgenic plants expressing the Flag-tagged MYC2 fusion protein were tested in ChIP assays with Flag-specific antibodies and DNA sequences from the *RD29B* and *RD29A* promoters.

**Fig. 9:**
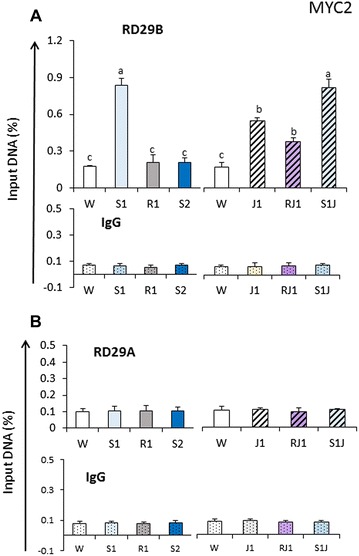
MYC2 distribution at the promoters of *RD29B* and *RD29A* in response to dehydration stress and to treatment with JA. ChIP–qPCR analysis of FLAG-MYC2 levels measured by FLAG-specific antibodies and the promoter regions of *RD29B* and at *RD29A* genes in transgenic plants expressing the FLAG-MYC2 fusion protein. **A** FLAG-MYC2 levels at *RD29B* in response to a first and a repeated dehydration stresses in plants untreated with JA. FLAG-MYC2 levels at *RD29B* upon exposure to JA, after the removal of JA and in response to the first dehydration stress, are shown on the right-hand side. Specific primers used for the recovered DNA are from the promoter (region 1) as indicated in Fig. 8. Background levels for immunoprecipitated samples with IgG for each amplicon are shown below; **B** FLAG-MYC2 levels at *RD29A* in response to dehydration stresses in plants untreated and treated with JA. Annotations, conditions, and IgG profiles are as described above for *RD29B*. The experiments were repeated three times, each with three replicates, and the representative experiments shown indicate the mean ± SE, *n* = 3 replicates. *Letters* above *bars* indicate significant difference among the treatments in the regions of interest (*p* < 0.05 according to Tukey’s multiple range test)

First, ChIP assays were conducted in plants that were not treated with JA. Significantly higher FLAG-MYC2 signal was measured in S1 at the *RD29B* promoter compared to the initial prestressed signal in W (Fig. 9A), indicating that MYC2 was recruited to *RD29B* in response to dehydration stress without a pre-treatment with JA. The recruitment is specific, as no increase in MYC2 was observed at *RD29A* in S1 (Fig. 9B). Interestingly, MYC2 returned to initial pre-stressed stressed levels during the recovery (in R1), suggesting that it dissociated from the promoter, in contrast to MED25 and TBP. Furthermore, the signal from MYC2 did not increase in S2, indicating that this transcription factor did not act directly on the *RD28B* promoter in S2. This result, together with the lower transcription from *RD29B* displayed in *myc2* mutants, created an apparent paradox (discussed further below).

Next, we measured MYC2 levels at the *RD29B* and *RD29A* promoters in response to the JA-treatment in water and in the response to the first dehydration stress. At the *RD29B* promoter, the presence of MYC2 increased during the exposure to JA (differences with water levels statistically significant, *p* < 0.05 according to Tukey’s multiple range test) and remained elevated (differences with water levels statistically significant, *p* < 0.05 according to Tukey’s multiple range test) during the recovery in the absence of JA. MYC2 levels further increased in response to dehydration stress, although the presence of MYC2 levels in S1J1 was not significantly different from the levels in S1 (Fig. 9A). The results suggested that MYC2 was recruited to *RD29B* by a dehydration stress and by exposure to JA. MYC2 levels in RS1J1 and in S2J1 were not measured because transcription in S2 was similar in JA-treated and JA-untreated plants. This implies that, regardless of MYC2 levels in these treatment points, transcriptional responses were not affected in S2 (see discussion further below).

MYC2 levels at the *RD29A* promoter did not significantly change in JA-pre-treated plants (Fig. 9B).

## Discussion

Treatment with JA induced expression of the Arabidopsis *PYL4* and *PYL5* genes [51], suggesting that expression of PYR/PYL/RCAR-ABA-regulated genes would be indirectly affected as well. Whether ABA-dependent genes could be regulated directly by a JA-signaling mechanism, however, has not been clarified. Our result here indicated that, although JA was unable to initiate directly the transcription from dehydration stress/ABA-regulated genes, JA-treatment primed specifically the *RD29B, RAB18, LTP3*, and *LTP4* genes linking, thus, the biotic stress JA-response pathway with these ABA-dependent [+/+] memory genes. Remarkably, however, JA potentiated transcription only in response to the first dehydration stress, supporting the idea that different mechanisms regulate memory genes’ transcription in S1 and in S2 [5, 21] (see further below).

### Molecular mechanism of the priming by JA

How pre-treatment with biotic stress hormones primes transcription of defense-related genes is an actively pursued current topic. Enhanced transcription after treatment with JA or SA/BTH has been linked to a rapid activation of hormones’ biosynthesis, accumulation of transcription factors and/or of the kinases/phosphatases regulating their activity [1, 3, 4, 6–8, 52, 53]. At the chromatin level, enrichment in H3K4me3 and in acetylated (H3K9, H4K5, H4K8, H4K12) histone marks at primed genes was implicated in their enhanced transcription upon subsequent attacks [13, 15, 22]. Small RNAs may play a role in the JA-mediated memory response as well [7]. However, what mechanism(s) lead to the accumulation of histone modifications or whether activated (Ser5P modified) Pol II is deposited at primed genes before transcription has not been established.

The most important contribution of this study is the elucidation of the molecular mechanism for the accumulation of Ser5Pol II and H3K4me3 at the primed dehydration stress responding genes facilitating their subsequently increased transcription. Our results here compellingly suggest that establishment of the Ser5Pol II/H3K4me3 marks at the JA-primed genes resulted from the JA-initiated/MYC2-mediated/MED25-dependent deposition of the basal transcriptional machinery to the genes before the initiation of their dehydration stress-induced transcription.

**Fig. 10:**
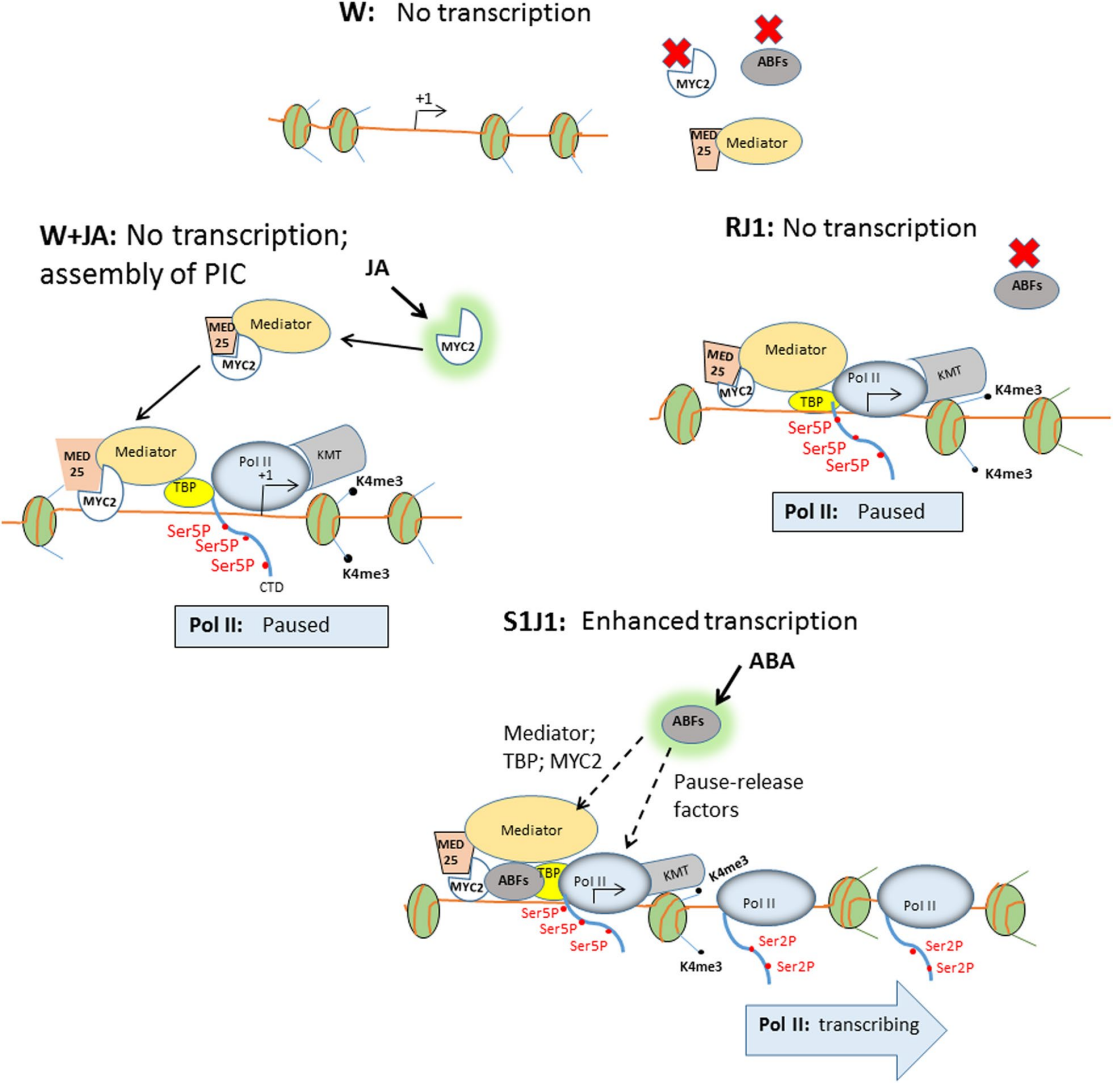
A model for the primed transcriptional responses from specific dehydration stress response genes. JA-primed dehydration stress response genes are not transcribed under watered conditions (W). The ABFs and MYC2 transcription factors are inactive. Treatment with JA activates MYC2 enabling it to bind the MED25 subunit of the complex and to recruit it to the specific promoters (W + JA). MEDIATOR stimulates the assembly of PIC and the installment of activated (Ser5-P) Pol II at the TSS marked with arrow [+1]. Ser5-P Pol II recruits histone K4 methyltransferases (KTM) establishing the H3K4me3 marks at 5′-end nucleosomes. Pol II remains in activated (paused, or stalled) state unable to initiate transcription before a dehydration stress; H3K4me3-modified nucleosomes and Pol II remain at the 5′-gene’s after removal of JA (RJ1) and in the absence of transcription. TBP, MEDIATOR, and MYC2 do not fully dissociate from the promoter in RJ1. Upon dehydration stress (S1J1), the levels of endogenous ABA increase activating the ABFs. The levels MEDIATOR and MYC2 also increase, but the nature of their interactions with the ABFs is unclear at present (shown with *dashed arrows*). H3K4me3 and Ser5P Pol II also increase and transcription is induced. The possibility that ABFs recruit key elongation factors to release stalled Pol II into active transcription is indicated by a *dashed line*

We found that treatment with JA increased H3K4me3 levels at dehydration stress response genes before active transcription and that elevated H3K4me3 persisted during the 22-h recovery (RJ1) when JA was no longer present (Fig. 3A). These H3K4me3 patterns occurred only at the superinduced [+/+] memory genes. Therefore, H3K4me3 functions as an epigenetic mark for JA-primed genes, regardless of whether their subsequent transcription is induced by biotic stress or by the ABA-mediated pathway. Activated but stalled Ser5P Pol II also accumulated at the JA-primed dehydration stress genes before transcription (Fig. 4A). Stalled promoter-proximal Pol II is emerging as a critical factor in chromatin modifications [37, 38]. In agreement, histone methyltransferases establishing the H3K4me3 marks are recruited by Ser5 Pol II to the 5′-ends of yeast, Drosophila, and Arabidopsis genes [2, 35, 54–56] providing a mechanism for the JA-dependent H3K4me3 occurrence observed here (Fig. 3A). The JA-dependent accumulation of Ser5Pol II and H3K4me3 at the memory genes before actual transcription may account for their enhanced transcription during the subsequent dehydration stress (higher in S1J1 compared to S1). A model of how the JA-mediated priming of specific ABA-dependent genes occurs is suggested (Fig. 10). It is based on the known abilities of MYC2 to bind directly MED25 in a JA-dependent manner [12, 32–34], of MEDIATOR to assemble PIC and to ensure phosphorylation of RNA Pol II CTD [45, 47, 48, 57, 58] and on the following results from this study: (1) the JA-priming effect depends on MED25 and on the transcriptional factor MYC2 as it is abrogated in *med25* and in *myc2* backgrounds (Figs. 5A, 7A); (2) MED25 accumulation at the promoters of primed genes before transcription is JA-dependent (Fig. 5E); (3) the Ser5P Pol II levels at the memory genes before initiation of transcription is JA-dependent (Fig. 4A), MED25-dependent (Fig. 6C) and MYC2 dependent (Fig. 8A); (4) the specificity of the process is determined by the transcription factor MYC2 (Fig. 7A, B); (5) overexpressed MYC2 mimics the JA-induced effects in wild-type plants, although the priming effects were stronger when the JA-signaling pathway was activated by exposure to exogenous JA (Fig. 7C). The enhanced *RD29B* transcription caused by constitutively expressed MYC2 in the absence of a JA-activated signaling mechanism is consistent with current understanding that in wild type, MYC2 is relieved from its repressed state by a JA-signaling mechanism allowing it to perform its molecular functions [29–31]; (6) MYC2 contributes to the transcription of the dehydration stress memory genes, as suggested by the lower transcript levels displayed in *myc2* background (Fig. 7A, Additional file 1: Figure S8). In addition, FLAG-MYC2 is also recruited to promoters of specific genes by a dehydration stress/ABA-mediated mechanism (Fig. 9A); (7) MYC2 remains slightly higher during RJ1 but dissociates from the promoters after the dehydration stress. This is in contrast to MED25, TBP, Ser5P Pol II, and H3K4me3, which are retained at *RD29B* during the water recoveries in RJ1 but also in R1 following S1; (8) transcription of the tested [+/+] memory genes occurs only after the dehydration stress, when the ABA-dependent transcription factors ABFs become activated.

### MED25/MEDIATOR complex at the dehydration stress/ABA-dependent genes

Our results implicated MED25 in facilitating the transcription of both the memory and non-memory dehydration stress genes tested here (Fig. 5A, B, Additional file 1: Figure S6), and ChIP analyses confirmed the presence of MED25 at their promoters in response to dehydration stress (Fig. 5C, D, Additional file 1: Figure S7). The results suggested that MED25 was taken to these promoters by transcription factors activated by dehydration stress/ABA-mediated mechanism(s). Earlier, it was shown that MED25 interacted with DREB2A, a transcription factor which induces the expression of *RD29A* and that MED25 negatively affected expression of ABI5 target genes during germination [12, 33, 59]. The ability of MEDIATOR to display both activating and repressive functions is due to its ability to form distinct complexes with related TFs in a tissue- and gene-specific manner [40, 46]. A mechanistic view of how oppositely functioning gene-specific MEDIATOR complexes form has been proposed [48].

It is specifically noted that the dehydration stress/ABA mechanism recruiting MED25 to the dependent response genes observed here is distinct from the JA-MYC2-mediated recruitment to the JA-primed dehydration stress memory genes. The higher presence of MED25 at primed genes in S1J1 (Fig. 5E, Additional file 1: Figure S7A) is consistent with an additional recruitment of MEDIATOR to the promoters by dehydration stress/ABA-activated transcription factors. Increased MED25-TBP-Ser5PolII-H3K4me3 levels correlate with the increased transcription in S1J1.

Deposition of the basal transcriptional machinery, of activated Ser5P, and of H3K4me3 marks in plants exposed to JA emerges as the main factor underlying the enhanced (primed) transcription of the specific dehydration stress response genes. Despite their presence, however, the JA-MYC2-MED25 mechanism is unable to induce transcription without the dehydration stress/ABA-mediated activation of the specific TFs, the ABFs [50, 60]. The questions of why no transcription takes place before dehydration stress and why Ser5P RNA polymerase II remains stalled at the JA-MYC2-MEDIATOR primed dehydration stress response genes, while transcription from JA-dependent target genes (i.e., *TAT3*) is induced (Fig. 2B), are part of the general questions of how Pol II stalling occurs, why it occurs at some genes but not at others, and how it is released into efficient elongation by specific transcription factors. Although the questions are still open, an emerging view is that MEDIATOR-deposited PIC and Pol II remain in a latent state at specific genes until key TFs (activated by a signaling pathway) appear. The ability of MEDIATOR to perform different functions, i.e., pre-initiation and/or elongation, at different promoters is attributed to its structural flexibility induced by the binding of specific TFs [45, 47, 48, 57, 58]. It is plausible then that the MEDIATOR complex recruited to promoters of ABA-dependent or JA-responding genes would respond differentially to ABA or to JA signals, which would determine whether Ser5P Pol II remains stalled or elongating. In addition to recruiting MEDIATOR-PIC complexes, ABA-activated ABFs might bring about additional factors to stimulate the release of Ser5P pol II into productive elongation (Fig. 10).

### The epigenetic marks are key to the JA-MYC2-MED25-induced responses to a repeated stress

Given that the JA-MYC2-MED25 mechanism specifically primed the superinduced [+/+] memory genes, it was somewhat surprising that their transcription was induced only in the response to the first dehydration stress (in S1J1), while the superinduced (memory) response in S2J1 was not significantly affected. We propose that stalled Ser5P Pol II and accumulated H3K4me3 uncoupled from transcription and serving as epigenetic marks for future enhanced transcription can explain this apparent paradox. In support, the presence of stalled Pol II is emerging as a critical factor in the rapid activation of metazoan gene expression upon induction [57, 59] and in Arabidopsis, retention of Ser5P Pol II and H3K4me3 after S1 is implicated in the superinduced transcription of the [+/+] memory genes in S2 [2]. It is conceivable, then, that the Ser5P Pol II and H3K4me3 remaining from the previous transcription (in S1) would contribute to their superinduced transcription in S2, regardless of whether expression in S1 has been primed by JA or not. Consequently, the JA-dependent accumulation of promoter-proximal Ser5P Pol II and H3K4me3 before dehydration stress is critical for potentiating the transcription of the [+/+] memory genes in their response to the first stress. The JA-signaling pathway, then, may not affect significantly transcription in S2 because Ser5P Pol II and H3K4me3 (formed in S1) are already available at the genes.

Therefore, transcription in S1 is necessary, partly, to establish Ser5P Pol II and H3K4me3 for the memory response in S2. However, the presence of these marks, alone, is not sufficient to achieve superinduced transcription, as transcript levels from primed genes in S1 are always lower than their levels in S2, despite the presence of Ser5P Pol II and H3K4me3 established by the JA-pathway. Clearly, other factor(s) generated in S1 and/or in S2 are required. This enigmatic ‘memory factor’ has not been identified yet. In previous studies, we have established that ABA is required but insufficient to induce transcription at the level achieved by dehydration stress in S2 [5]. The TFs ABF1, ABF2, and ARF3, and the histone methyltransferase ATX1 affected strongly the transcription in S1 and in S2 but the memory, albeit attenuated, was not erased in their absence [2]. MYC2 and MED25 also played roles in S1 and in S2, as transcript levels were lower in the *myc2* and *med25* backgrounds but transcript levels in S2 were always higher than in S1 (Figs. 5A, 7A, Additional file 1: Figure S6A, Figure S8A).

MYC2 contributes to the transcription of the dehydration stress memory genes and was found at the *RD29B* promoter in S1 without a pre-treatment with JA (Figs. 7A, 9A), indicating that MYC2 is recruited to specific genes in response to dehydration stress. In future studies, it will be interesting to determine whether this recruitment is achieved by an ABA-mediated mechanism or depends on endogenously generated JA resulting from the dehydration stress [61, 62].

Given that the expression of the *MYC2* gene is induced only in S1 but is significantly down in S2 [21] and that MYC2 was not recruited to the promoter of *RD29B* in S2 after dissociating in R1 (Fig. 9A), the requirement for MYC2 in the high-level transcription of the memory genes in S2 (Fig. 7A) creates another apparent paradox. We speculate that the role of MYC2 in S2 is indirect, i.e., regulating the expression of a factor(s) in S1 that would stimulate superinduced transcription in S2. It will be important to study this question in the future as it could help revealing the ‘memory factor(s)’ that are responsible for the superinduced transcription in S2. The available evidence, however, suggests that MYC2 affects the *RD29B* transcription in S1 and in S2 by different mechanisms.

### Possible biological relevance of the JA-priming of dehydration stress memory genes

Here, we focus on the question of whether the specifically exerted JA potentiation of superinduced dehydration stress/ABA-dependent genes observed here may have any biological relevance for the plant. This aspect of the JA- and ABA-pathways’ interactions is different from their well-known interactions in response to biotic and abiotic environmental stresses or in diverse developmental processes [for recent relevant papers and reviews, see [63–69] and ref. therein]. Despite the large number of shared genes known to be co-regulated by the JA- and ABA-signaling pathways [24, 25, 70–73], there is no evidence, to our knowledge, of JA-response genes that are primed by JA to provide a stronger response to ABA; furthermore, none of the dehydration stress/ABA-dependent genes studied here has been recognized or defined as a target of a JA-mediated mechanism. A possible reason is the inability of JA to influence immediate transcriptional responses from these dehydration stress/ABA-dependent genes. Whether such priming is biologically relevant may be established only by specifically designed and targeted experiments. Possible clues, however, may be suggested from available transcriptome data of multiply stressed plants [9].

Whole-genome analysis of Arabidopsis plants exposed to three rounds of dehydration stress treatments has identified about 320 genes with [+/+] memory behavior, while about 2000 genes induced by the first stress provided similar transcriptional responses to each dehydration stress (non-memory genes) [9]. In addition, more than 300 JA-associated genes, including genes for key JA biosynthesis enzymes (AOC1, AOC2, OPR, LOX2), for components of the core JA-signaling pathway (JAZ genes), and for numerous JA-mediated targets, respond to a single dehydration stress but do not provide a response to a second stress. These genes, referred to as ‘revised response’ memory genes, belong to a different memory category than the [+/+] memory genes studied here (see Table 2 and discussion in [9]) and whether their transcription under dehydration stress is primed by JA is currently unknown.

The distinctive feature of the [+/+] memory genes, setting them apart from the rest of the dehydration stress response genes, is that they produce significantly more transcripts under a repeated encounter with the stress than they produced during the first. *RD29B, RAB18, LTP3*, and *LTP4* are members of this gene subset, and here we found that they generate more mRNAs also in their response to the first dehydration stress if they were exposed to JA before encountering the stress. A potential biological significance of this transcriptional behavior may be considered on the basis of encoded functions. GO analysis of proteins encoded by [+/+] memory genes has identified numerous enzymes for the synthesis of osmolytes and for detoxification, of molecular chaperons, of proteins for repair mechanisms, for membrane maintenance and preservation of membrane fluidity [9]. Overall, the signature feature of the [+/+] memory genes is synthesis of proteins involved in specific cell-protective functions upon repeated exposures to dehydration stress by superinducing production of mRNAs encoding these functions. In agreement, *RD29B, RAB18, LTP3*, and *LTP4* encode dehydrins preventing stress-induced membrane damage and proteins maintaining membrane fluidity, respectively. Their specific priming by JA, then, may be interpreted as a step toward increased production of protective proteins should the plant encounter a subsequent dehydration stress.

Specifically emphasized also is that the ability of JA to potentiate transcription from the ABA-dependent genes reported here does not imply a particular environmental stress or physiological condition associated with increased presence of JA. Whether resulting from a pathogen, from wounding, herbivore, senescence or abiotic stresses, a JA-triggered mechanism selectively potentiated ABA-dependent genes that encode specific cellular/membrane protective functions. More studies are needed to reveal whether/how endogenous JA levels may affect performance of ABA-response genes. The biologically important implications of such studies are particularly important in the context of the different physiological responses provided by plants to a combination of stresses compared to their responses to a single stress [69, 71, 72, 74–76]. Adjustment of genes’ expression under recurring stress episodes allows the plant to optimize its responses, its interactions with other signaling pathways, and to provide a more robust stress response while reducing the costs of the state of preparedness [73, 76–78].

## Conclusions

In conclusion, although the biotic stress hormone (JA) was unable to induce directly the transcription of dehydration stress response genes in Arabidopsis, pre-exposing plants to JA substantially potentiated (primed) the transcription upon a subsequent dehydration stress from a specific subset of the response genes. The molecular mechanism of this priming is based on the JA-triggered deposition of the basal transcriptional machinery to the promoters of ABA-dependent memory genes. JA-activated binding of MYC2 to MED25 recruits the MEDIATOR complex to specific dehydration stress response promoters, where the MEDIATOR recruits TBP (PIC) and facilitates the phosphorylation at Ser5 of the Pol II CTD; on its part, Ser5PPol II contributes to the establishment of H3K4me3 at 5′-end nucleosomes. All these events take place before the occurrence of active transcription. Accumulation of H3K4me3 at the primed genes affecting subsequent transcription defines them as key epigenetic marks in the priming event. MEDIATOR is critical in potentiating ABA-dependent memory genes linking the JA-priming and the dehydration stress response pathways at the transcriptional level. A dehydration stress/ABA mechanism can recruit the MED25 (MEDIATOR) complex to the promoters of response genes as well and that this process is independent of the JA-triggered mechanism. Apparently, two separate mechanisms can recruit MED25 to dehydration genes’ promoters. Although less efficiently, transcription still occurs in the *med25* background, suggesting that the role of MED25 is mainly to facilitate their transcription. The specific priming of superinduced dehydration stress memory genes suggests that cell/membrane protective functions would be more strongly expressed upon a first encounter of dehydration stress, had the plant experienced also stress from JA.

## Methods

### Plant growth and treatments

Wild-type *A. thaliana* (Col-0 background) and mutant plants (described below) were grown in potting soil in growth rooms at 22 °C with a 12-h light photoperiod and light intensity of 180 μmol m^−2^ s^−1^. Three-week-old plants were removed from pots, their roots carefully washed for any remaining soil and placed in humid chambers with roots immersed in drops of distilled water, for an overnight recovery. Plants were grown in mesh-covered pots, and all manipulations were done by handling the meshes (see Fig. 1C) to minimizing damage of plant tissues. This first 22-h recovery in water after removal from soil is annotated as the initial prestressed water treatment state (W). JA-treatment is achieved by moving meshes containing experimental plants into Petri dishes and placing roots in a few drops of 50 µM me-JA solution (Cayman Chemical, USA) for 2 h (J1); control sample of untreated plants was similarly manipulated by moving plants in Petri dishes and placing roots in water. After the JA-treatment, roots were washed twice with tap water to remove residue JA, followed by 22-h recovery in humid chamber, roots in water (RJ1). The second day, JA-pre-treated and untreated seedlings were blotted on paper towel to remove water and air-dried for 2 h (S1 or S1J) followed by a 22-h recovery in humid chamber (R1 or R1SJ). On the third day, same dehydration stress was applied (S2 or S2J1). A summary diagram of the treatments is shown in Fig. 1B. JA and dehydration stress treatments were performed from 10 am to 12 pm, and samples for quantitative PCR measurements were collected immediately after. The mutants and transgenic Arabidopsis lines used in study were previously described: *myc2*-*2* (Salk_040500); *pMYC2:MYC2*-*FLAG*/*jin1*-*8* [79]; *med25*-*4* [80]; *35S*::*MED25*-*HA* [34].

### Reverse transcription and real-time PCR

Mixed leaves from ten 3-week-old plants were used for RNA extraction. Total RNA isolation and reverse transcription with oligo (dT)_18_ (18418-012; Invitrogen, Carlsbad, CA) were performed as previously described [23]. The amounts of individual genes were measured with gene-specific primers by real-time PCR analysis with a cycler IQ real-time PCR instrument (Bio-Rad, Hercules, CA) and SYBR Green mixture (Bio-Rad, Hercules, CA). The relative expression or amount of specific genes was quantitated with the $2^{{ - {\varDelta \varDelta }C_{\text{t}} }}$ calculation [81] according to the manufacturer’s software (Bio-Rad, Hercules, CA), where ΔΔ*C*_t_ is the difference in the threshold cycles and the reference housekeeping gene, which was ubiquitin for expression analyses or relative to input DNA for chromatin immunoprecipitation assays. The specific primers used are shown in Additional file 2: Table S1. Statistical analyses were performed using the SPSS 22.0 (SPSS) package to compare the qRT-PCR data. Multiple comparisons between wild type and mutant plants across the different treatments were determined by factorial ANOVA with differences among means tested at *p* = 0.05 using a Tukey’s post hoc test. Other differences between JA-treated and JA-untreated plants were determined using Student’s *t* test.

### Chromatin immunoprecipitation (ChIP) assays

Aboveground tissues from ten, 3-week-old wild type and mutant Arabidopsis were fixed in 1 % formaldehyde under vacuum. Fixed tissues were homogenized, and chromatin was isolated and sonicated as described [2, 82]. The solubilized chromatin was immunoprecipitated by adding corresponding antibody for an overnight incubation at 4 °C. The specific antibodies used for: trimethyl-H3K4 (ab1012, Abcam, Cambridge, MA, Lot: GR50173); Ser5P Pol II (ab5131, Abcam, Cambridge, MA, Lot: GR57107); anti-histone H3 (ab1791, Abcam, Cambridge, MA, Lot:GR50118); anti-TATA binding protein (ab52887, Abcam, Cambridge, MA, Lot: 347607); anti-flag (F3165, Sigma); or anti-HA (Ref 11867423001, Roche, Indianapolis, IN, Lot: 13006600). The antibody–protein complexes were isolated by binding to protein A or protein G magnetic beads (Invitrogen, 1101D or 1107D). The washed beads were heated at 65 °C for 8 h with proteinase K to reverse the formaldehyde cross-linking and digest proteins. The sample was then extracted with phenol/chloroform and the DNA precipitated in ethanol and re-suspended in water. The recover percentage of co-immunoprecipitated DNA was calculated by normalizing the amount of a target DNA fragment against that of a genomic fragment (inputs). The specific primers used are shown in Additional file 3: Table S2. Statistical analyses were performed as said above.

## Additional files

10.1186/s13072-016-0057-5 Supplementary figures S1–S9.
10.1186/s13072-016-0057-5 List of primers used for qRT analysis.
10.1186/s13072-016-0057-5 List of primers used for ChIP-qPCR analysis.

## Abbreviations

Ser5P Pol II: DNA-dependent RNA polymerase II phosphorylated at serine 5 of its C-terminal domain
JA: jasmonic acid
ABA: abscisic acid
SA: salicylic acid
BTH: benzo-(1,2,3)-thiadiazole-7 carbothioic acid
RD29A/B: response to dehydration 29A, response to dehydration 29B
RAB18: responsive to ABA
COR15: cold responding15
TAT3: tyrosine aminotransferase3
LTP3/4: lipid transfer protein 3, lipid transfer protein 4
JAZs: jasmonate zim-domain proteins
TPL: TOPLESS
MYC2: member of the basic helix–loop–helix transcription factor family
MED25: subunit 25 of the MEDIATOR complex
PIC: pre-initiation complex
ChIP-qPCR: chromatin immunoprecipitation–quantitative PCR
qRT-PCR: quantitative reverse transcription PCR
