# Supplementary material for: Molecular mechanism of the priming by jasmonic acid of specific dehydration stress response genes in Arabidopsis
Source: Epigenetics Chromatin. 2016 Feb 24;9:8. doi: 10.1186/s13072-016-0057-5 (PMC4766709; doi:10.1186/s13072-016-0057-5)
Supplement: Supplementary file 1 — 10.1186/s13072-016-0057-5 Supplementary figures S1–S9. [file 13072_2016_57_MOESM1_ESM.pdf]

Supplementary Figures

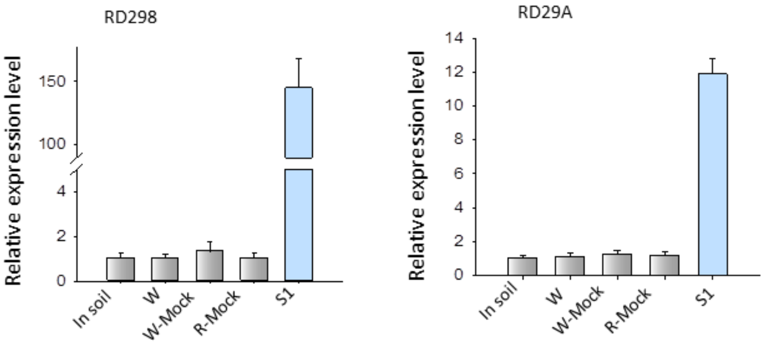

Figure S1 Transcript levels from *RD29B* and *RD29A* in soil and under water treatments

Transcript levels measured by real-time quantitative RT-PCR from *RD29B* and from *RD29A* genes in leaves of plants while still in soil and during treatments under water conditions as indicated in Figure 1B. qPCR data are normalized versus *ACT8*. Error bars represent the SE from the 3 biological replicates.

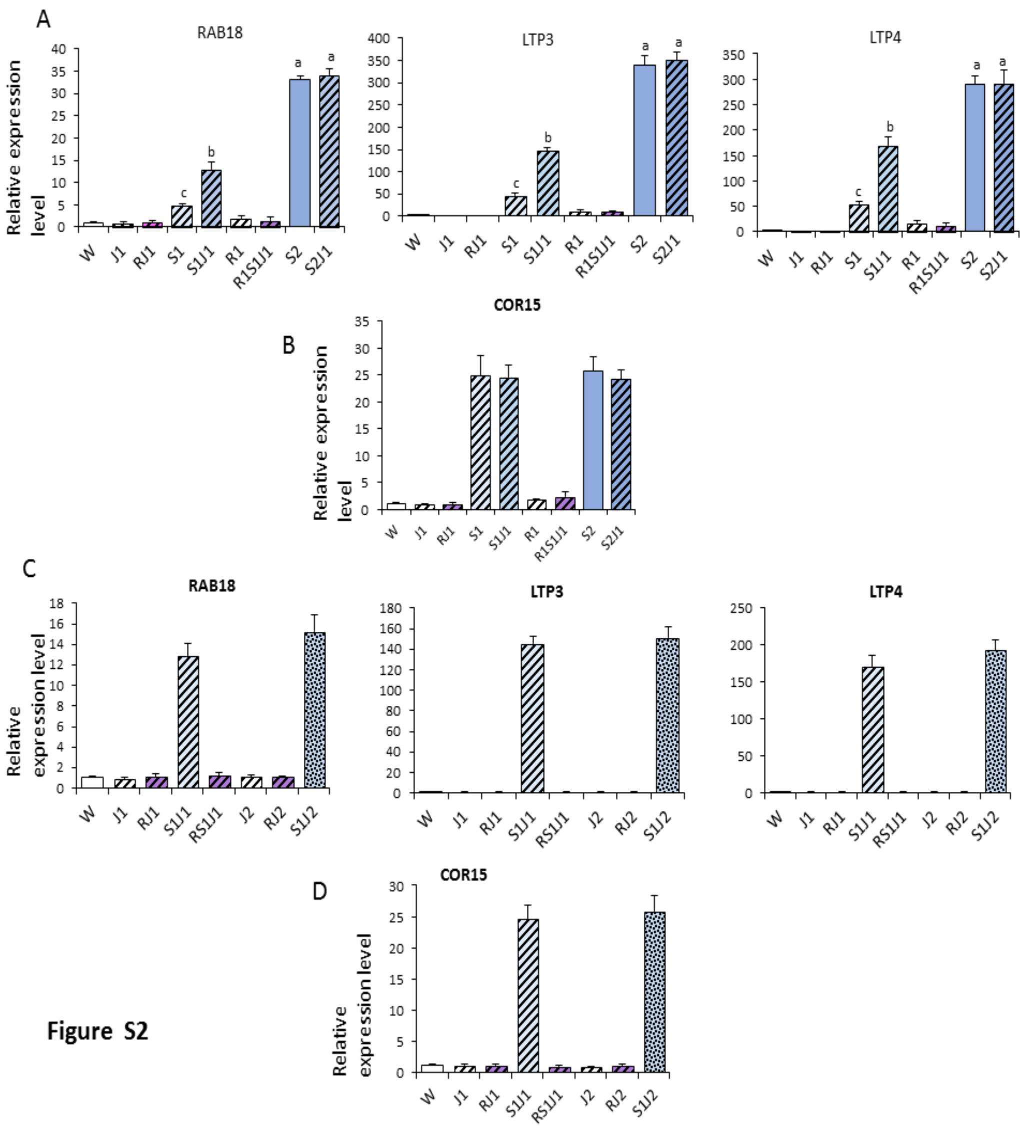

Figure S2 Transcription patterns of *RAB18, LTP3, LTP4,* and *COR15* in response to dehydration stress after single or repeated exposures to JA

A-B) Transcript levels measured by real-time quantitative RT-PCR from *RAB18*, *LTP3*, *LTP4,* and *COR15* genes in response to two consecutive exposures to dehydration stress with and without pre-treatment with JA. Annotations under columns indicate treatment points as specified in Figure 1B in text; **C-D)** Transcript levels from memory *RAB18, LTP3, LTP4,* and the non-memory *COR15* genes, respectively, in response to dehydration stress after experiencing two exposures to JA (S1J1 and S1J2). Data have been normalized with *ACT8*. Experiments were repeated at least three times, each with three qPCR measurements. Results are the average of 3 independent experiments, error bars indicate the standard error of the mean. Letters above the error bars indicate significant differences between stress responses, $p < 0.05$ according to Tukey's multiple range tests.

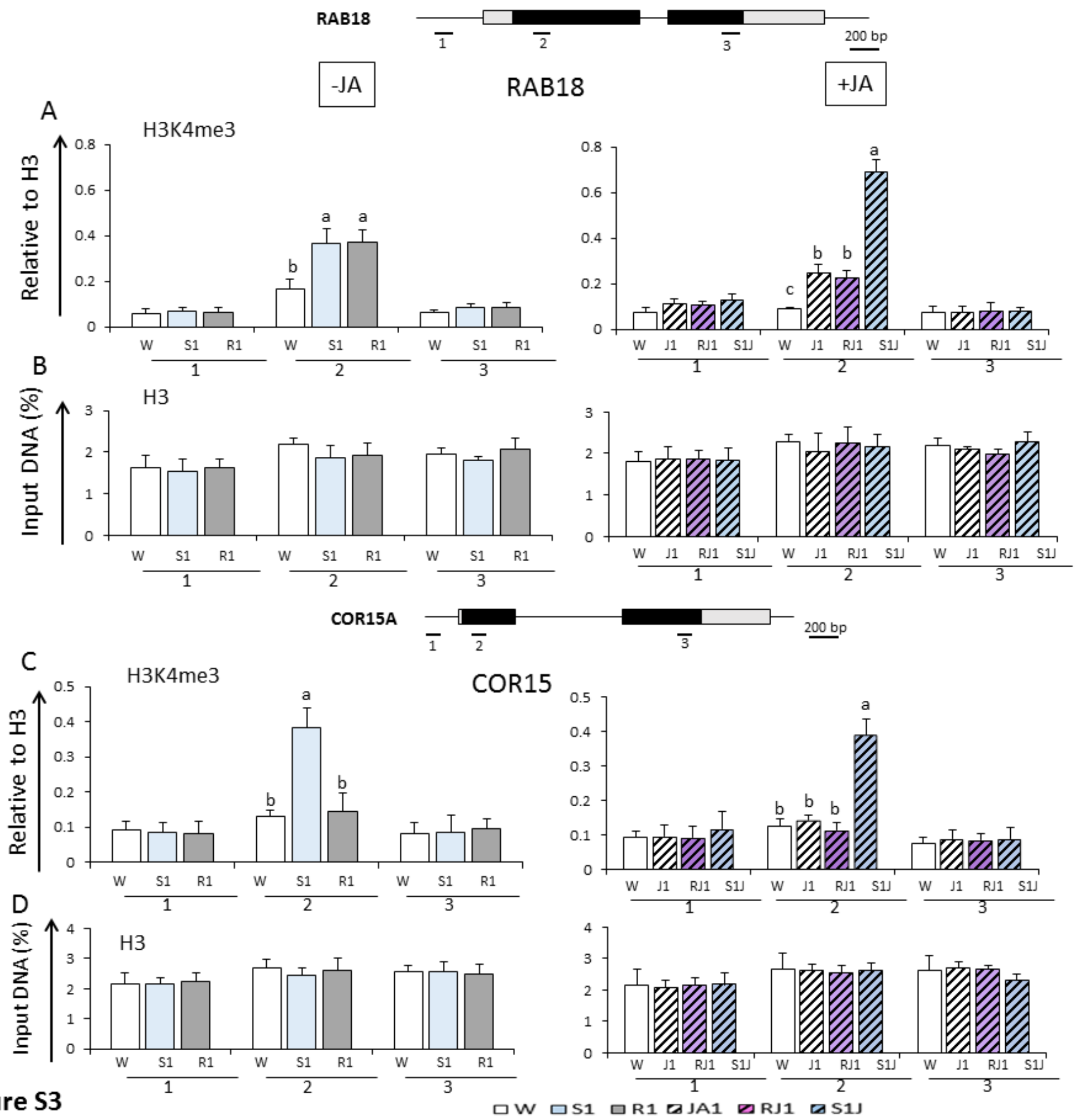

Figure S3

Figure S3 H3K4me3 and Histone H3 distribution patterns at *RAB18* and *COR15* genes in response to dehydration stress and to treatment with JA

The levels of histone H3K4me3 and of histone H3 distributions determined by ChIP–qPCR assays with specific antibodies under various treatments annotated as indicated in Figure 1B. *RAB18* and *COR15* provide supporting information for the H3K4me3 profiles at a memory and non-memory gene, respectively. **A)** H3K4me3 levels at three different locations along *RAB18* as indicated by the gene diagram on top. Data from JA-treated and untreated samples are shown in the same scale. H3K4me3 data were normalized versus values for histone H3; **B)** Histone H3 distribution determined by ChIP-qPCR with histone H3 specific antibodies and DNA recovered from the same amplicons shown below. Data from JA-treated and untreated samples are shown in the same scale; **C)** H3K4me3 levels at *COR15* at the regions indicated by the gene diagram on top. H3K4me3 data were normalized versus values for histone H3 at the same locations. Data from JA-treated and untreated samples are shown in the same scale; **D)** Histone H3 distribution

determined by ChIP-qPCR with histone H3 specific antibodies and DNA recovered from the *COR15* regions indicated in the gene diagram on top. Data from JA-treated and untreated samples are shown in the same scale. Numbers below bars indicate probed regions, as illustrated by the schematic diagram of the genes on top, where region (1) corresponds to promoter upstream from the TSS, region (2) is immediately downstream of TSS, where the K3K4me3 peak accumulates, and region (3) corresponds to downstream 3'-end sequences. Untranslated regions (grey box), exons (dark box), and introns (thin lines between exons). Experiments were repeated 3 times each with three RT-qPCR measurements, and the representative experiment shown indicates the mean ± SE, n = 3 replicates. Different letters above bars indicate significant difference among the treatments in the region of interests ($P < 0.05$ according to Tukey's multiple range test).

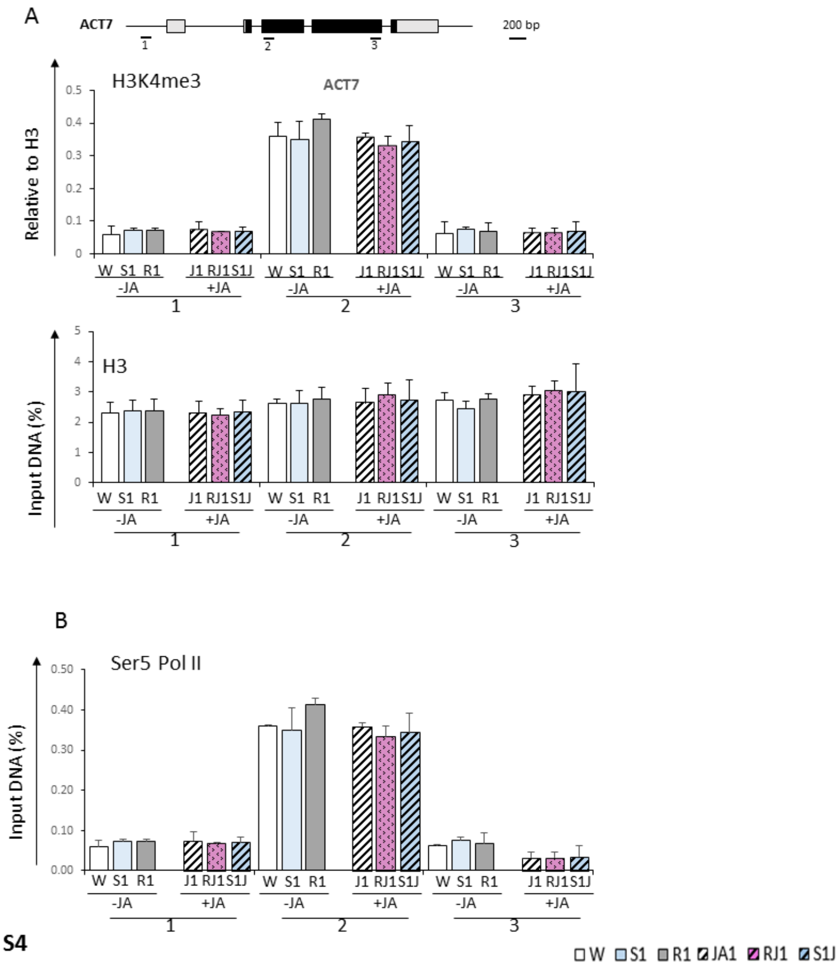

Figure S4

Figure S4 H3K4me3, Histone H3 and Ser5 Pol II distribution patterns at the *ACT7* gene in response to dehydration stress and to treatment with JA

A) H3K4me3 levels at three different locations along *ACT7* as indicated by the gene diagram on top. Data from JA-treated and untreated samples are shown in the same scale. H3K4me3 data were normalized versus values for histone H3; **B)** Histone H3 distribution determined by ChIP-qPCR with histone H3 specific antibodies and DNA recovered from the same amplicons shown below. **C)** Ser5 Pol II levels at *ACT7* at the regions indicated by the gene diagram on top. Experiments were repeated at least three times each with three RT-qPCR measurements, and the representative experiment shown indicates the mean ± SEM, n = 3 replicates.

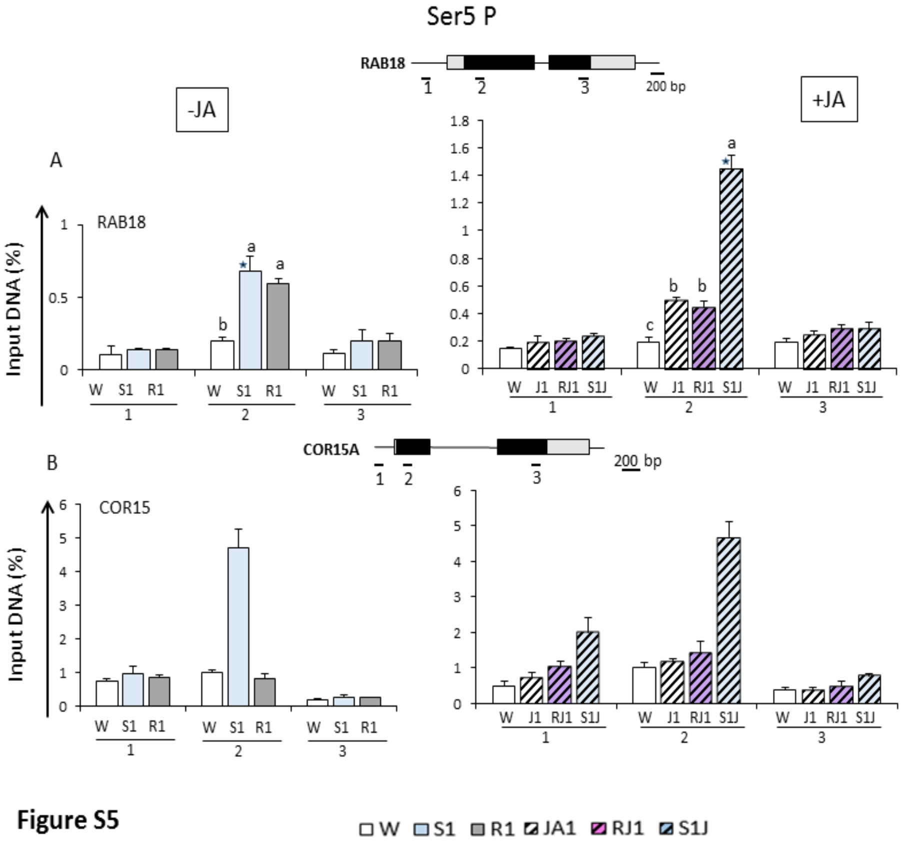

Figure S5 Ser5P Pol II at the memory *RAB18*, the non-memory *COR15* in response to dehydration stress and to treatment with JA

Ser5P Pol II levels measured by ChIP–qPCR assays with specific antibodies under the various treatments, as annotated in Figure 1B. Schematic diagrams of the genes are shown on top and all annotations are as in Figure 3 above. Region 2 is where the peak of Ser5P Pol II accumulates. **A)** Ser5 Poll II levels measured by ChIP-PCR at the indicated regions of *RAB18*. Data from JA-treated and untreated samples are presented in the same scale and asterisks at S1 and S1J1 treatments indicate significant differences according to student t-test ($P<0.01$); **B)** Ser5 Poll II levels measured by ChIP-PCR at the indicated regions of *COR15;* Experiments were repeated at least three times each with three RT-qPCR measurements, and the representative experiment shown indicates the mean ± SEM, n = 3 replicates. Different letters above the bars indicate significant differences between treatments ($p < 0.05$, according to Tukey's multiple range test).

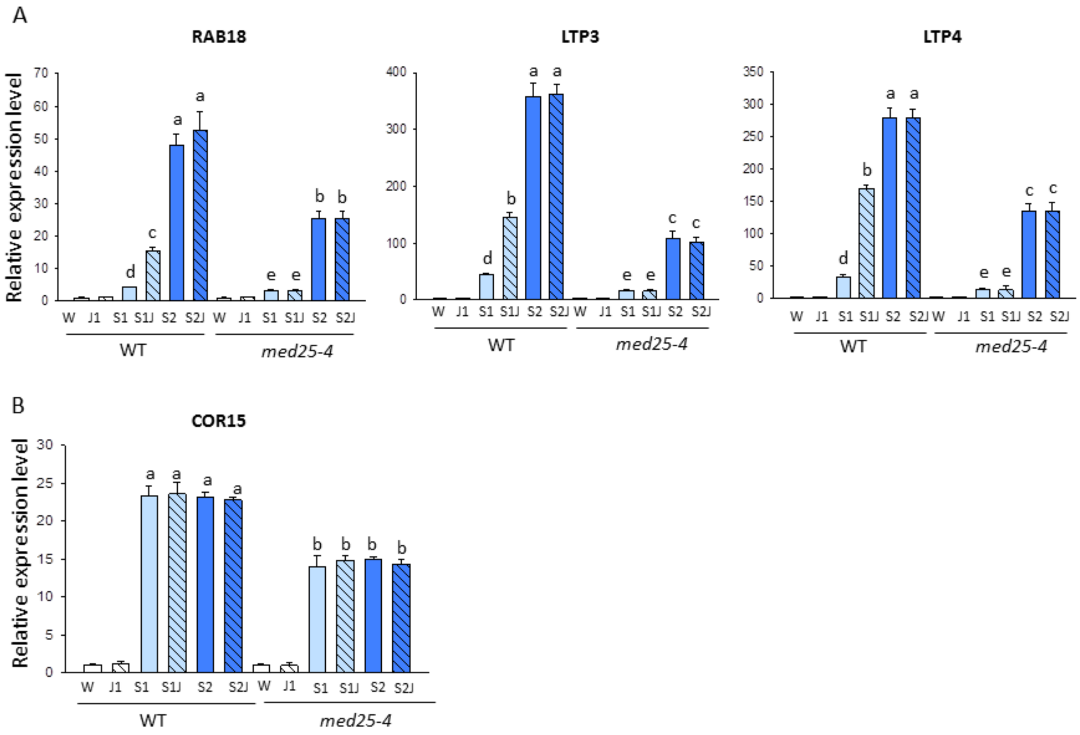

Figure S6 Transcript levels of the memory and non-memory genes under dehydration stress in *med25* background with and without JA-treatment

A) Transcript levels of memory *RAB18*, *LTP3*, and *LTP4* genes and **B)** the non-memory *COR15* gene in Col-0 and in *med25* mutant backgrounds measured by real-time quantitative RT-PCR under the various treatment conditions, as indicated. Data have been normalized with *ACT8*. Results are the average of three biological replicates; error bars indicate the standard error of the mean. Different letters above the bars indicate significant differences between drought treatments ($p < 0.05$, according to Tukey's multiple range test).

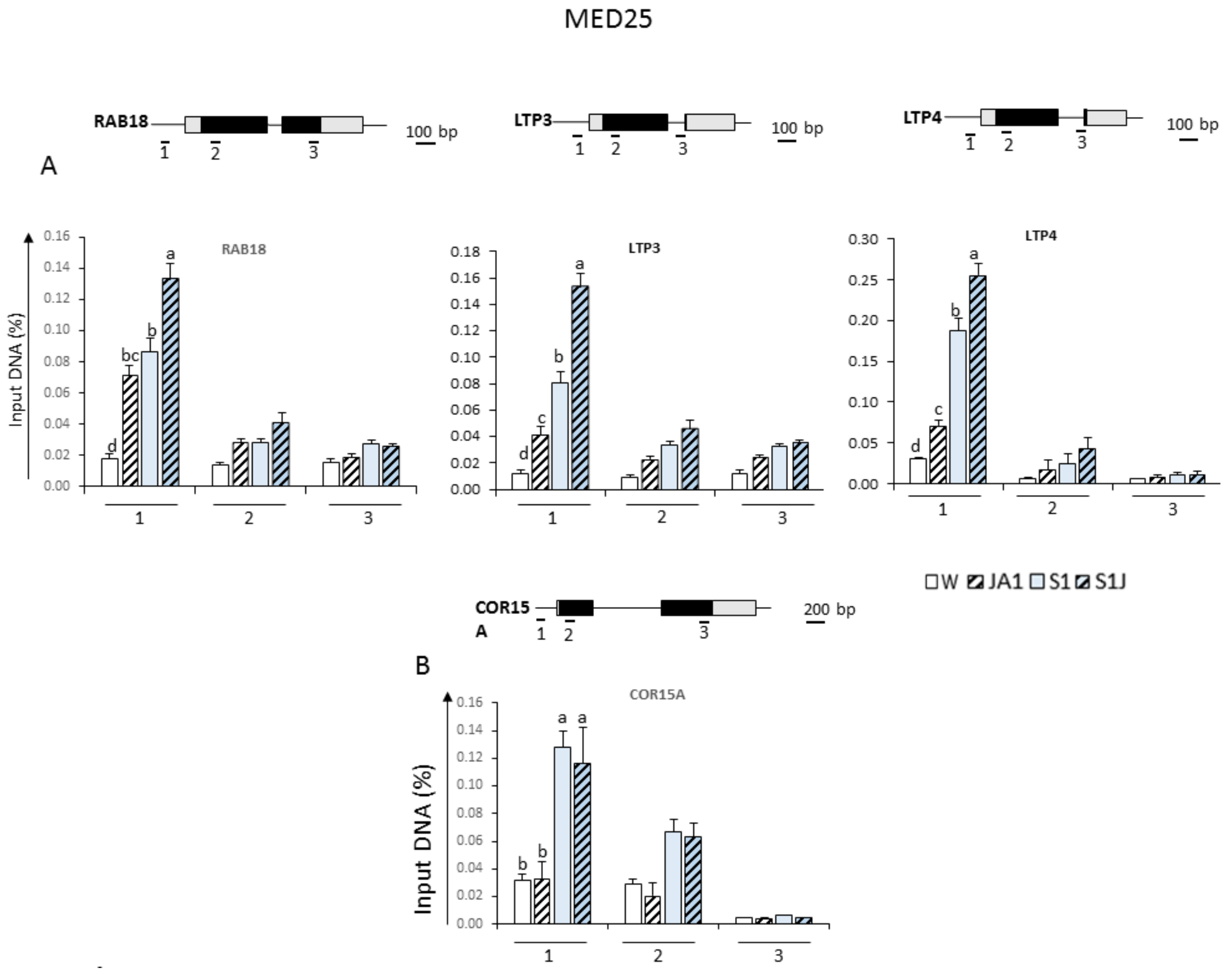

Figure S7 Distribution profiles of MED25 at the *RAB18, LTP3, LTP4,* and *COR15* genes

A) Distribution profiles of HA-tagged MED25 determined by ChIP–qPCR assays with antiHA-antibodies and DNA sequences from the tested regions of the memory *RAB18, LTP3, LTP4* genes and **B)** from the non-memory *COR15* gene as indicated on the respective gene structure diagrams. Values from the same regions of JA-untreated and JA-treated samples are shown in parallel. Experiments were performed 3 times, and the ChIP values are mean ± SEM (n = 3). For each point, letters above bars indicate significant difference among the treatments in the regions of interest, $p < 0.05$ according to Tukey's multiple range test).

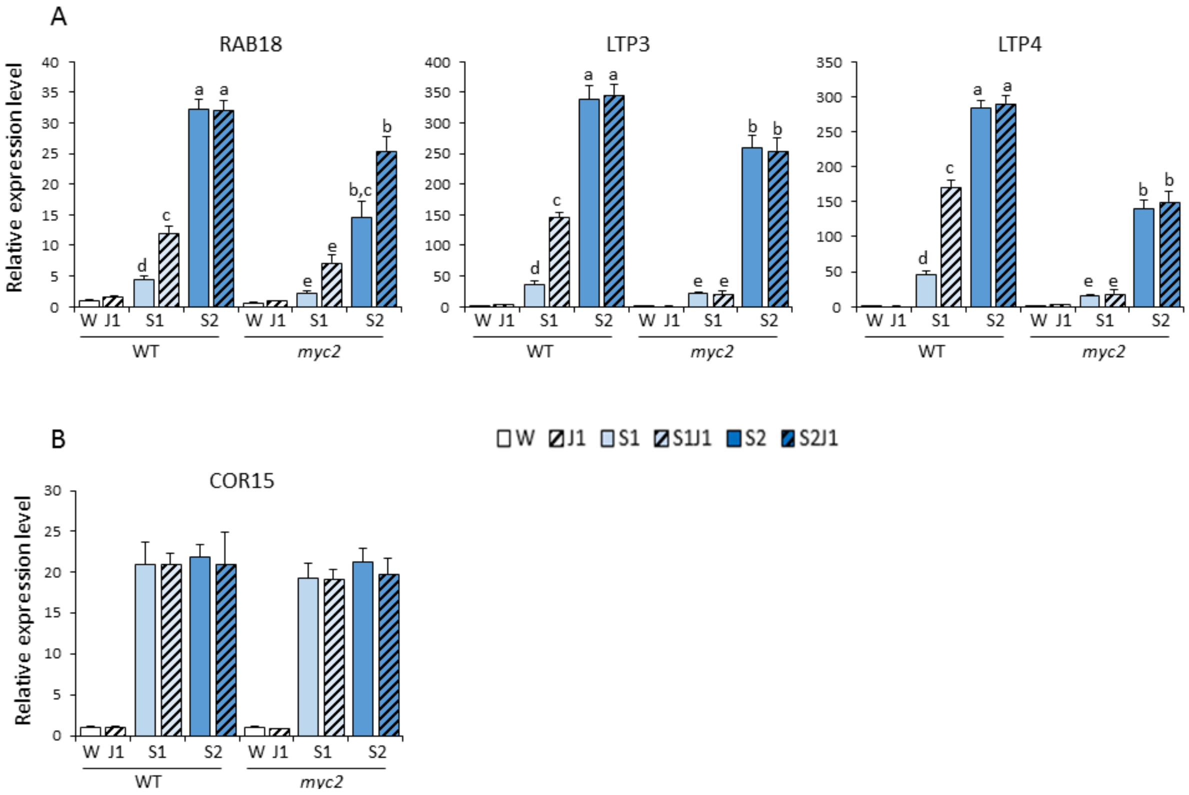

Figure S8 Transcript levels of *RAB18, LTP3, LTP4,* and *COR15* genes in wild type and *myc2* backgrounds in response to dehydration stress with and without treatment with JA

A) Transcript levels of *RAB18, LTP3, LTP4,* and **B)** of *COR15A* measured by real-time quantitative RT-PCR in wild type and in *myc2* backgrounds in response to a repeated dehydration stress and JA-treated and untreated plants. Data are normalized with *ACT8*. Experiments were repeated at least three times. The experiments shown indicate the mean ± SEM (n = 3). Letters above bars indicate significant difference in transcript levels among the drought treatments ($p < 0.05$ according to Tukey's multiple range test).

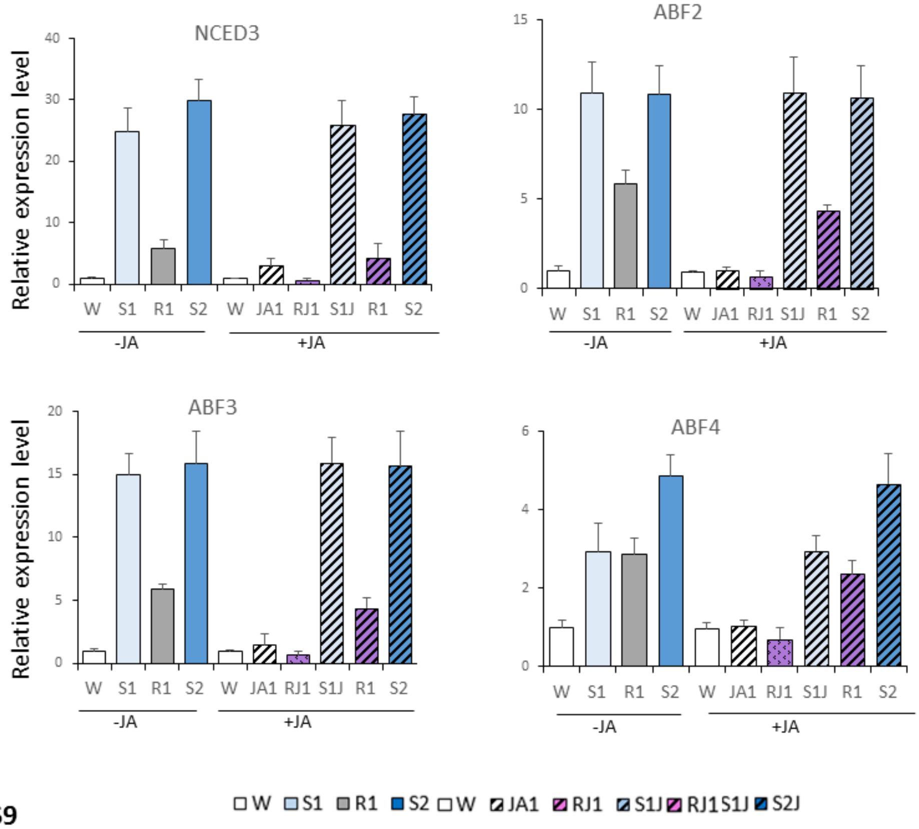

Figure S9

Figure S9 Transcriptional responses by *NCED3* and the key ABFs TFs to a repeated dehydration stress with or without pre-treatment with JA

Transcript levels of *NCED3*, *ABF2*, *ABF3* and *ABF4* to repeated dehydration stresses without exposure to JA and after pre-treatment with JA. Transcript levels were measured by real-time quantitative RT-PCR and data normalized with *ACT8.* Results are the average of three independent experiments, each with two replicates. Error bars indicate the standard error of the mean ± SEM (n = 3).
