# Supplementary material for: Molecular mechanism of the priming by jasmonic acid of specific dehydration stress response genes in Arabidopsis
Source: Epigenetics Chromatin. 2016 Feb 24;9:8. doi: 10.1186/s13072-016-0057-5 (PMC4766709; doi:10.1186/s13072-016-0057-5)
Supplement: Supplementary file 2 — 10.1186/s13072-016-0057-5 List of primers used for qRT analysis. [file 13072_2016_57_MOESM2_ESM.pdf]

Supplementary Table 1: List of primers used for qRT analysis

Genes	Sequence (5'-->3')
RD29A	AGGAACCACCACTCAACACA
	ATCTTGCTCATGCTCATTGC
RD29B	ACGAGCAAGACCCAGAAGTT
	AGGAACAATCTCCTCCGATG
RAB18	GCACAATACAACGACCGAATGCGA
	GGTGGCCGTTAAGCTTCGAACAAT
COR15A	CAGATGGTGAGAAAGCGAAA
	CCCTACTTTGTGGCATCCTT
ACT8	TCAGCACTTTCCAGCAGATG
	CTGTGGACAATGCCTGGAC
LTP3	AACGGTGTGCATAGTTGCAT
	TGACTCCTGCACAACATGAA
LTP4	GCTTTCGCTTTGAGGTTCTT
	TGTGCCACATGTTATTGCTG
ABF2	GGGTTGACTAGACAAGGTTCG
	ATCCATGTTCATTGACCCAA
ABF3	TGGTGGAATTGGGAAAGATT
	CAACAGCTAACCCACCAATG
ABF4	TAGGAGGTGGTGGTCATCCT
	CCGGTCCACCTAGTGTGTT
TAT3	GTCCCAAGAAACCCGAATCT
	GCTTCGTGCAGAAATCAAAA
NCED3	ATGTTTCATCTGCGCTTCAC
	CGCTCTCTGGAACAAATTCA
ACT8	TCAGCACTTTCCAGCAGATG
	CTGTGGACAATGCCTGGAC
