# Supplementary material for: Molecular mechanism of the priming by jasmonic acid of specific dehydration stress response genes in Arabidopsis
Source: Epigenetics Chromatin. 2016 Feb 24;9:8. doi: 10.1186/s13072-016-0057-5 (PMC4766709; doi:10.1186/s13072-016-0057-5)
Supplement: Supplementary file 3 — 10.1186/s13072-016-0057-5 List of primers used for ChIP-qPCR analysis. [file 13072_2016_57_MOESM3_ESM.pdf]

Supplementary Table 2. List of primers used for ChIP-qPCR analysis

Genes		Sequence (5'-->3')
RD29B	Region 1	CGTAGAGAGCAACTGGCTGA
		ATTCTGACACGTACGATGCG
	Region 2	AATGGAGTCACAGTTGACACG
		GGATGGTGAATTCTGATTGGT
	Region 3	TGGAAGTGACGGTTGAGAAG
		ACCGCTCCTTTAACTTTCCC
RAB18	Region 1	TGAGCTTTCATGTCGATACCA
		AGGAGGAGCATTACGTGTCC
	Region 2	TCCTTGTGGAGTTGCTCTTG
		GGACTGAAGGCTTTGGAACT
	Region 3	TAGCCACCAGCATCATATCC
		AAGGAGGGAGGAGGAAGAAG
LTP3	Region 1	TGGCCACAGTTAATTAAAAGCA
		GACGGACACGTGTACCCAAC
	Region 2	TCAGTCGATGCTGCAATCTC
		TCCTGCACAACATGAAGGTG
	Region 3	TTGATCTAGAGACCATGAATTATTTTT
		CGACGTAAGCTTCCATTTCA
LTP4	Region 1	CATGCCGTCTGATTTAATGC
		GAAAGGTGGTCCAATGGAAA
	Region 2	CCCATCATCATCTCCCACTT
		TTGCTCTTCTCTTTTGGGTGA
	Region 3	CGACATCATTTGCCTGAAGA
		CAAAGCCATCAAGACAAACAAA
RD29A	Region 1	ACCGACATCAGTTTGAAAGAAA
		TGGTGTGACGTCAAAGTCATT
	Region 2	TCACTAAACATGGACAAAGCAA
		TGCATCGATCACTTCAGGTT
	Region 3	CTCCATCAAGAAGCCATGAA

		GGCGAATACTCGTTTCTTCC
COR15A	Region 1	CCGAGTTTCTGTTCGTTCTTT
		CAATTTCATGGCCGACCT
	Region 2	GCCATACCAGTGAGAACAGC
		CCTCCTTTCATTTCCAAACAA
	Region 3	CAGATGGTGAGAAAGCGAAA
		CCCTACTTTGTGGCATCCTT
ACT7	Region 1	ATTTGAACGATGTCCGAACC
		GAGCCGTGACTGATGGTTAC
	Region 2	CGTTTCGCTTTCCTTAGTGTTAGCT
		AGCGAACGGATCTAGAGACTCACCTTG
	Region 3	GCTGACCGTATGAGCAAAGA
		GATCCTCCGATCCAGACACT
